# Biophysical subsets of embryonic stem cells display distinct phenotypic and morphological signatures

**DOI:** 10.1371/journal.pone.0192631

**Published:** 2018-03-08

**Authors:** Tom Bongiorno, Jeremy Gura, Priyanka Talwar, Dwight Chambers, Katherine M. Young, Dalia Arafat, Gonghao Wang, Emily L. Jackson-Holmes, Peng Qiu, Todd C. McDevitt, Todd Sulchek

**Affiliations:** 1 The G. W. Woodruff School of Mechanical Engineering, Georgia Institute of Technology, Atlanta, GA, United States of America; 2 The Wallace H. Coulter Department of Biomedical Engineering, Georgia Institute of Technology & Emory University, Atlanta, GA, United States of America; 3 School of Biological Sciences, Georgia Institute of Technology, Atlanta, GA, United States of America; 4 School of Chemical & Biomolecular Engineering, Georgia Institute of Technology, Atlanta, GA, United States of America; 5 Gladstone Institute for Cardiovascular Disease, San Francisco, CA, United States of America; 6 Department of Bioengineering and Therapeutic Sciences, University of California San Francisco, San Francisco, CA, United States of America; 7 The Parker H. Petit Institute for Bioengineering and Bioscience, Georgia Institute of Technology, Atlanta, GA, United States of America; University of Texas at Austin Dell Medical School, UNITED STATES

## Abstract

The highly proliferative and pluripotent characteristics of embryonic stem cells engender great promise for tissue engineering and regenerative medicine, but the rapid identification and isolation of target cell phenotypes remains challenging. Therefore, the objectives of this study were to characterize cell mechanics as a function of differentiation and to employ differences in cell stiffness to select population subsets with distinct mechanical, morphological, and biological properties. Biomechanical analysis with atomic force microscopy revealed that embryonic stem cells stiffened within one day of differentiation induced by leukemia inhibitory factor removal, with a lagging but pronounced change from spherical to spindle-shaped cell morphology. A microfluidic device was then employed to sort a differentially labeled mixture of pluripotent and differentiating cells based on stiffness, resulting in pluripotent cell enrichment in the soft device outlet. Furthermore, sorting an unlabeled population of partially differentiated cells produced a subset of “soft” cells that was enriched for the pluripotent phenotype, as assessed by post-sort characterization of cell mechanics, morphology, and gene expression. The results of this study indicate that intrinsic cell mechanical properties might serve as a basis for efficient, high-throughput, and label-free isolation of pluripotent stem cells, which will facilitate a greater biological understanding of pluripotency and advance the potential of pluripotent stem cell differentiated progeny as cell sources for tissue engineering and regenerative medicine.

## Introduction

**Fig 1:**
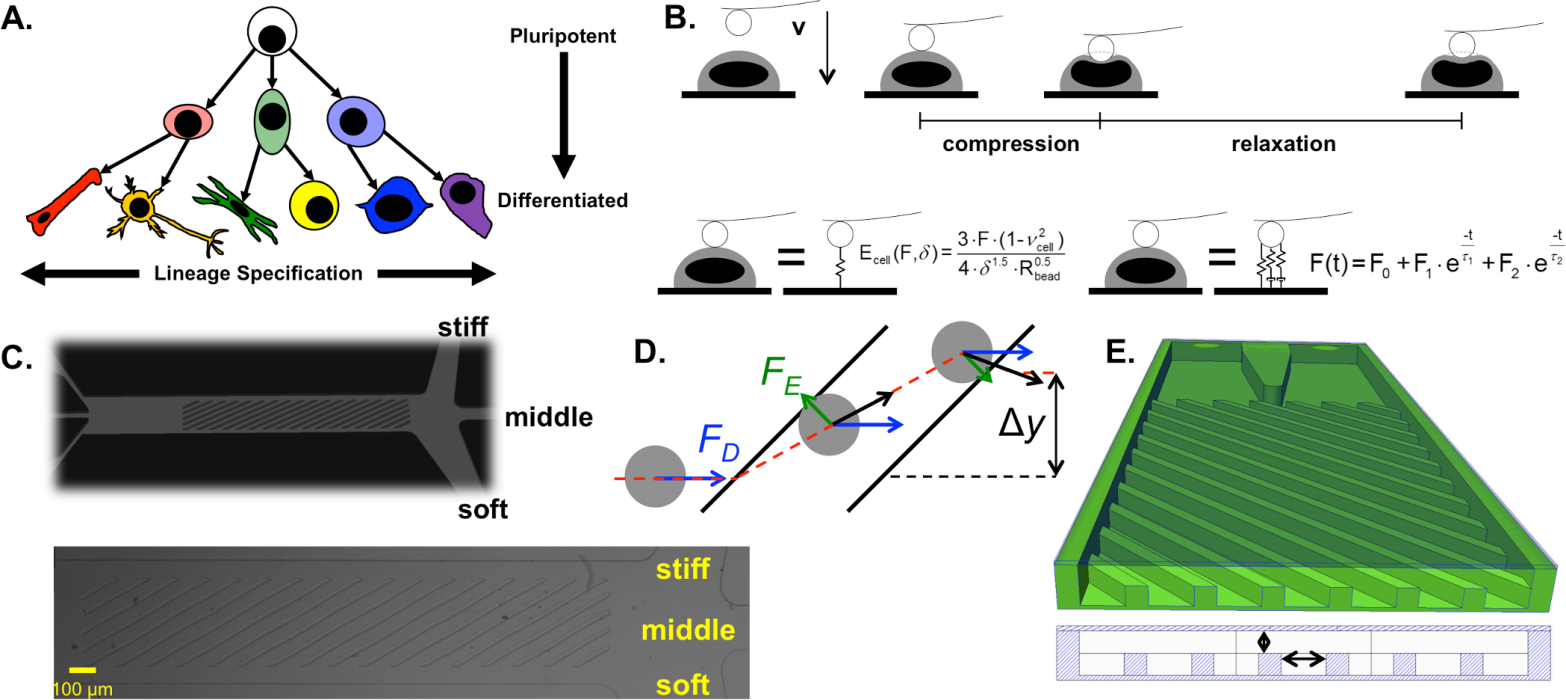
Biophysical characterization and sorting. (A) Lineage specification, as illustrated by hue, changes based on the set of phenotypes to which a given cell can differentiate. Cell maturity, as illustrated by tone, is lowest for an embryonic stem cell (light) and increases during specification to a terminally differentiated cell (dark). (B) Cellular mechanics parameters were assessed by atomic force microscopy using a beaded cantilever. The cantilever was translated toward the cell until a 5 nN trigger was registered, completing the compression region of the force curve; subsequently, the relaxation of the cell was measured over 10 s. The compression region was fit to the Hertzian model to calculate the cellular Young’s modulus, *E*_cell_. A two-Maxwell-element viscoelastic model was fit to the relaxation portion of the force curve, yielding two viscoelastic time constants, *τ*_1_ and *τ*_2_. (C) To sort cells based on biophysical parameters, a microfluidic device with diagonal ridges was employed. (D) As a cell approaches each diagonal ridge, the ridge compresses the cell, creating an elastic force (*F*_*E*_, green arrows). The cell is also exposed to a ridge-generated secondary flow that imposes a hydrodynamic drag force (*F*_*D*_, blue arrows). The net force, and therefore the trajectory (red dashed line with net displacement *Δy*) of each cell, is stiffness-dependent. (E) The critical geometrical parameters were the gap size, *h*, and the ridge spacing, *r*. The gap size determines the strain imposed on a cell of a given size. The time a cell takes to travel between ridges (the inter-ridge time), which can be tuned via the overall flow rate or the inter-ridge spacing, affects the degree to which cell trajectory depends on viscoelastic relaxation.

Tissue-engineered organs and regenerative medicine therapies are estimated to require >10^7^ cells of one or more prescribed cell types [1], which is difficult to achieve using autologous cell sources. Embryonic stem cells (ESCs) hold great potential as scalable, phenotype-specific “cell factories,” but progress is hampered by the two-fold challenge of directing cell fate commitment to specific lineages and controlling the maturity of a particular cell type (Fig 1A).

ESC cultures can be quite heterogeneous and typically contain not only colonies of pluripotent ESCs, but also outgrowths of fibroblast-like differentiated progeny [2]. Even within putative pluripotent ESC colonies, the expression of pluripotency markers can be heterogeneous [2]. However, controlling both lineage specification and maturity in ESC-derived cell populations is paramount for tissue engineering and regenerative medicine. Lineage specification must be controlled to obtain well-defined cell populations with sufficient phenotypic purity, which are required to generate functional tissue-engineered organs and efficient cell therapies [3]. Controlling maturity is also important, as retention of the pluripotent phenotype can cause dangerous teratomas [4], whereas differentiation results in reduced proliferation rates of stem cell progeny [5], which can limit the efficacy of cell therapy [6].

To address the challenges of controlled linage specification, extensive efforts have been made to engineer the cellular microenvironment through directed differentiation protocols. However, current protocols are generally low yield, often with less than 50% target phenotype, or time-consuming, typically requiring several weeks or more. A low yield hampers the ability to use the cell population for applications such as tissue engineering, which requires a well-defined ratio of specific cell types to create an organ of interest that closely matches *in vivo* physiology. A complementary method of phenotype control is to select target cell types from a heterogeneous population, which requires an understanding of the cell subsets that exist for each selection basis, such as cell morphology, gene expression, and/or protein expression.

Biomolecular subsets of stem cells have been well studied [7,8], but cell identification based on biomolecular expression is limited by the inconsistent and poorly understood expression of gene and protein markers for specific phenotypes. Biomarker expression can be transient, and the absence or presence of multiple markers is typically required to accurately define cell phenotype. To address this problem, we and others [9–12] have proposed cellular mechanics parameters as additional factors to help identify phenotype. Mechanical parameters offer the potential for both non-terminal probing of live cells and high-throughput sorting at the single-cell level. Indeed, a recent study [13] demonstrated that although the stiffness of populations of adipose-derived stem cells did not change during adipocyte differentiation, individual cells that were positive for peroxisome proliferator receptor gamma, an adipocyte marker, were significantly softer than cells that did not express the marker. However, in general, biophysical subsets of stem cells and their relationships with potency, lineage specification, and molecular expression are not well studied.

Therefore, the objective of this study was to understand the biological characteristics of distinct biophysical subsets of ESCs. The results indicate that pluripotent cells are softer than differentiating cells and that the soft biophysical subset of partially differentiated cells displays a similar signature to pluripotent cells, with regard to cell mechanics, morphology, and gene expression. The present work serves as a step toward high-throughput enrichment of specified ESC-derived cell phenotypes or depletion of unwanted pluripotent cells for tissue engineering and regenerative medicine applications.

## Methods

### Cell culture

Mouse ESCs (D3 cell line, ATCC, Manassas, VA) were cultured in growth media (15% fetal bovine serum [Atlanta Biologicals, Atlanta, GA], 2 mM L-glutamine [ThermoFisher, Waltham, MA], 1x MEM non-essential amino acid solution [Mediatech, Herndon, VA], 0.1 mM 2-mercaptoethanol [ThermoFisher], 100 U/mL penicillin, 100 *μ*g/mL streptomycin, and 0.25 *μ*g/mL amphotericin [Mediatech] in Dulbecco’s modified Eagle’s medium [Sigma-Aldrich, St. Louis, MO]) on polystyrene Petri dishes treated with 0.1% gelatin (Millipore, Billerica, MA). Media was changed every other day, and cells were passaged at approximately 70% confluence.

To support pluripotency, growth media was supplemented with 1.1 U/*μ*L leukemia inhibitory factor (LIF; Millipore). Starting from pluripotent colonies, as identified by rounded morphology, differentiation was induced by culturing the ESCs in -LIF growth media. Alternatively, media containing bone morphogenetic protein 4 (BMP-4) was used to direct differentiation to the mesoderm lineage, as previously described [14]. Briefly, ESC aggregates were formed by centrifugation into Aggrewells™ (Stem Cell Technologies, Vancouver, BC, Canada; 500 cells/well), maintained on a rotary orbital shaker platform at 65 rpm, and differentiated in mesoderm induction media (10 ng/mL BMP-4 [R&D Systems, Minneapolis, MN], 2 mM L-glutamine [ThermoFisher], 100 U/mL penicillin, 100 *μ*g/mL streptomycin, and 0.25 *μ*g/mL amphotericin [Mediatech] in ESGRO complete basal media [Millipore]) for up to 10 days.

Mouse embryonic fibroblasts (MEFs; SCRC-1008, ATCC) were cultured in growth media (15% fetal bovine serum [Atlanta Biologicals], 100 U/mL penicillin, 100 *μ*g/mL streptomycin, and 0.25 *μ*g/mL amphotericin [Mediatech] in Dulbecco’s modified Eagle’s medium [Sigma-Aldrich]) on tissue culture polystyrene Petri dishes. Media was changed every other day, and cells were passaged at approximately 70% confluence.

### Preparation of cell suspensions

For microfluidics experiments that employed cell staining to distinguish pluripotent from differentiating ESCs, pluripotent +LIF ESCs were stained with 500 nM CellTracker™ Green CMFDA (ThermoFisher) and differentiating -LIF cells were stained with 5 *μ*M CellTracker™ Red CMTPX dye (ThermoFisher), using the manufacturer’s protocol. For the remaining microfluidics experiments and all biophysical characterization experiments, cell dyes were not used due to concerns that staining can change cell mechanics [15]. Prior to biophysical characterization or microfluidic sorting, cells were detached from the gelatin-coated dishes using a solution of 0.05% trypsin and 0.53 mM ethylenediaminetetraacetic acid (EDTA; Sigma-Aldrich), dissociated by trituration, and pelleted by centrifugation.

### Biophysical characterization

Approximately 100,000 cells were plated on glass dishes coated with 1.5 *μ*g/cm^2^ poly-L-lysine and immobilized during 16–24 h incubation at 37°C. Immediately prior to probing, non-adherent cells were removed by washing the dish twice with phosphate-buffered saline (PBS) containing magnesium and calcium, with 1 min incubation for each wash step. To simplify the tip-cell contact geometry, one 5.5 *μ*m polystyrene bead was attached to each tipless silica nitride cantilever (Bruker Probes, Camarillo, CA) using two-part epoxy and dried overnight. Mechanical properties of individual cells were obtained from force-indentation curves recorded with an atomic force microscope (Asylum Research, Santa Barbara, CA) with an integrated optical microscope (Nikon, Melville, NY) on a vibration isolation table. Atomic force microscopy is summarized in Fig 1B. The Sader calibration method [16] was used to obtain cantilever spring constants (k = 6–14 pN/nm) based on the thermal vibration of the cantilever. The cantilever probe was visually aligned with the cell center and translated to indent the cell with a velocity of 2 *μ*m/s until a force trigger of 5 nN was reached. To examine the cell relaxation under compression, the cantilever dwelled at the surface of the compressed cell for 10 s while the cellular relaxation response was recorded.

To calculate the cellular Young's modulus, the Hertzian contact model was fit to the compression segment of the force-indentation curve over the applied force range of 2.5–4.75 nN, where the Young's modulus was largely independent of the indentation. The cells were assumed to be incompressible (cellular Poisson's ratio = 0.5). The Young's modulus of each cell was calculated as the average of 3 independent measurements.

To calculate the viscoelastic properties of the cells, the spring-damper model was fit to the relaxation segment of the force-time curve, using the Maxwell–Wiechert model to calculate the viscoelastic time constants [17]. Two Maxwell elements were chosen to best fit the data. The fast and slow viscoelastic time constants were designated as τ_1_ and τ_2_, respectively. The viscoelastic properties of each cell were calculated as the averages of 3 independent measurements.

### Morphology characterization

To calculate the spread cell size and shape factors for each cell, ImageJ (National Institutes of Health, Bethesda, MD) was employed to manually draw a polygon around each cell from the phase contrast images captured during atomic force microscopy. Size histograms for suspended cells were obtained using a Multisizer Coulter Counter (Beckman Coulter, Brea, CA), which was calibrated using polystyrene microspheres of known sizes (Polysciences, Warrington, PA).

### Cytoskeletal staining

One day prior to reaching the differentiation time points of 0–6 days, cells were plated on glass coverslips coated with poly-L-lysine and immobilized during 16–24 h incubation at 37°C. The cells were fixed with 4% PFA for 10 min and stored in PBS at 4°C. The cells were then permeabilized with 0.1% Triton X-100 for 10 min, blocked with 5% BSA in PBS for 1 h, and incubated with 1:40 Phalloidin 488 (ThermoFisher), which stains F-actin, and 1:5000 Hoescht 33342 (ThermoFisher), which stains DNA, in PBS for 30 min. The coverslips were washed 3 times with 0.1% Tween-20 in PBS for 5 min, mounted to glass slides with Prolong Gold (ThermoFisher), cured overnight, and sealed. Confocal images were obtained using a Plan-ApoChromat 63X/1.4 NA oil objective on a Axiovert 200M inverted microscope (Zeiss, Oberkochen, Germany) with a UltraView Vox spinning disk (Perkin Elmer, Waltham, MA) and C11440-22C camera (Hamamatsu, Hamamatsu City, Japan). Volocity imaging software (Perkin Elmer) was used to acquire the raw images, perform uniform contrast adjustments, and generate the final images, which represent the maximum intensity projections of the z-stacks. Each cell was scored manually into one of three morphological types.

### Microfluidic device fabrication and sample preparation

Microfluidic sorting devices with 2 or 3 outlets were fabricated as previously described [18,19]. A reusable SU-8 mold (MicroChem, Westborough, MA) containing the device features was formed using standard two-step photolithography on a silicon wafer. A mixture of polydimethylsiloxane pre-polymer and curing agent (PDMS; 10:1 v:v; Sylgard 184, Dow Corning, Auburn, MI) was used for replica molding with curing at 60°C for 6 h. After curing, the 1 mm inlet holes and 3 mm outlets holes were punched, enabling each outlet to serve as a reservoir for cell collection. The PDMS devices were treated with air plasma using a plasma cleaner (Harrick Plasma, Ithaca, NY) and bonded to glass slides to form the microfluidic chips. After plasma bonding, the channels were incubated at 60°C for 1 h to further strengthen bonding.

The sorting buffer consisted of 87.5 nL/mL Tween-20, 40 *μ*g/mL EDTA, and 1 mg/mL bovine serum albumin (BSA) in a 3:7 (v:v) mixture of Percoll (GE Life Sciences, Pittsburgh, PA) and PBS lacking magnesium and calcium. Prior to sorting, the pH of the buffer was adjusted to 7.4 and the buffer was filtered with a 0.22 *μ*m pore filter. To reduce the fraction of cells that either floated to the top or sunk to the bottom of the syringe over time, the ratio of Percoll to PBS was tuned such that the buffer density matched the average cell density, thereby limiting local fluctuations in cell concentration and keeping the cell concentration constant during sorting. The ratio was optimized using density centrifugation with various Percoll:PBS ratios. Maintaining the buffer at 4°C and including BSA, EDTA, and Tween-20 facilitated the maintenance of a single-cell suspension. The inlet flow rates were controlled using syringe pumps (Harvard Apparatus, Holliston, MA).

For the cell system described in this study, the gap size, which controls the strain experienced by each cell (see Fig 1C–1E), and the overall flow rate, which controls the inter-ridge relaxation time for each cell, were optimized to maximize separation. To optimize the total flow rate, videos were recorded using a high-speed camera (Vision Research, Wayne, NJ) during individual sorts of unstained cells after either 0 or 5 days of differentiation. For various total flow rates, cells reaching each outlet were counted manually using a hemocytometer. In all flow rate optimization studies, the fraction of cells reaching the stiff outlet was negligible compared to the fractions of cells reaching the soft and middle outlets.

### Biophysical subset characterization

Cells were manually collected from the outlet reservoirs and periodically transferred to uncoated polystyrene Petri dishes containing growth media. Prior to characterization, cells were transferred to a tube and pelleted. Before mechanical characterization, cells were plated on poly-L-lysine-coated glass dishes and immobilized during 16–24 h incubation at 37°C. The mechanics and morphology of single cells taken from each biophysical subset were measured by atomic force microscopy and phase contrast microscopy. To prepare samples for gene expression measurements, 100 cells from each sample replicate were dispensed into cell collection buffer (CellsDirect™ 2x reaction mix [ThermoFisher] containing 1 U/*μ*L SUPERase In™ RNase inhibitor [ThermoFisher] to prevent degradation) using a FACSAria Fusion™ cell sorter (BD Biosciences, San Jose, CA).

### Primer design and gene expression analysis

Primers were obtained from Invitrogen, as listed in S1 Table, and resuspended in DNA suspension buffer (Teknova, Hollister, CA). Primer pairs were designed using Primer3 [20,21] and validated for RT-qPCR using the CellsDirect™ One-Step qRT-PCR kit (ThermoFisher) and a StepOne Plus with SYBR Green detection chemistry (ThermoFisher). LinRegPCR software [22–24] was used to baseline-correct the amplification traces and measure the amplification efficiencies. Melt curves were used for preliminary screening of primer pairs for primer dimers and multi-product reactions, and all primer products were validated for length and specificity using gel electrophoresis on a 1% agarose gel run in 1.5x TAE buffer.

The lysed cells were mixed with the pooled set of primers (normalized to 500 nM), SuperScript® III RT Platinum® Taq Mix, and nuclease-free water. A thermocycler was used to convert RNA to cDNA, with reverse transcription occurring at 50°C for 15 min, followed by reverse transcriptase inactivation and Taq activation at 95°C for 2 min. To amplify the cDNA using the pooled primers, the sample was exposed to 20 cycles of 15 s at 95°C and 4 min at 60°C. The cDNA samples were stored at 4°C. cDNA samples were then processed with exonulcease I to remove any unincorporated primer and diluted 5-fold. 3 *μ*L of each sample and primer mix was prepared for the FLEX Six IFC chip (Fluidigm, South San Francisco, CA). Finally, a Biomark™ HD (Fluidigm) was used to thermal cycle the chip 30 times and read the amplification via EvaGreen® fluorescence.

Threshold fluorescence signals and the corresponding threshold cycle values were obtained using the Real-Time PCR Analysis software package (Fluidigm) with automatic detector thresholds. Initial target DNA concentrations, *N*_*0*_, were calculated as previously described [25], using $N_{0}=\frac{t}{\epsilon^{C_{t}}}$, where *t* is the threshold fluorescence signal for each target, *C*_t_ is the threshold cycle for each sample, and ε is the reaction efficiency, assessed as the mean efficiency calculated using LinRegPCR software. Samples with a z-score magnitude greater than 3.5 were excluded, as previously described [22].

Initially, the expression levels of *Rn18s*, *Pax6*, and *Myf5* were also measured. However, the extremely high abundance of *Rn18s* caused exponential amplification by cycle 2, resulting in highly variable threshold cycle readings. The low abundance of *Myf5* and *Pax6* precluded PCR amplification in most samples. Therefore, *Rn18s*, *Pax6*, and *Myf5* were removed from the analysis.

### Statistics and figure generation

In total, paired stiffness-morphology data were obtained for 359 cells. Data were grouped by differentiation method and day of differentiation; 80 of the cells were undifferentiated (day 0), 162 were differentiated by LIF removal in monolayer (day 1, n = 29; day 2, n = 30; day 3, n = 30; day 4, n = 29; day 5, n = 28; day 6, n = 16), 59 were differentiated by LIF removal in embryoid body format (day 6, n = 29; day 10, n = 30), and 58 were differentiated by BMP-4 treatment in embryoid body format (day 6, n = 29; day 10, n = 29). Paired stiffness-viscosity-morphology data were available for 30 of the undifferentiated cells and for all 162 cells differentiated by LIF removal in monolayer.

To discern statistically significant differences, bootstrapping ANOVA was performed using a custom code in MATLAB (MathWorks, Natick, MA). The ANOVA p-value was compared to *α*_*ANOVA*_ = 0.05; for significant ANOVA p-values, Holm's adjustment was applied to the pairwise p-values and compared to *α*_*post-hoc*_ = 0.1, as previously reported [12]. The presence of univariate and multivariate outliers precluded the use of MANOVA to compare the relative impact of the session, passage number, day of differentiation, differentiation method, and differentiation format on cell stiffness; therefore, all subsequent analyses and experiments were confined to the monolayer, LIF removal differentiation method (N = 242) and one-way bootstrapping ANOVA was employed to assess changes to cell mechanics during differentiation. Spearman's rank correlations were assessed by first using JMP statistical software (SAS Institute, Cary, NC) to obtain coefficients and raw p-values and subsequently using a custom Excel spreadsheet (Microsoft, Redmond, WA) to apply Holm's p-value adjustment (*α* = 0.1) and plot the resulting color matrices. For differentiation studies, pluripotent cells were coded as 0 and differentiating cells were coded as 1. For biophysical subset studies, the soft subset was coded as -1, the middle subset was coded as 0, and the stiff subset was coded as 1. When comparing the day of differentiation and the differentiation state, the Pearson's correlation coefficient is +1, as expected; however, the Spearman's rank correlation coefficient is lower due to the method JMP invokes to break ties.

Beeswarm plots and semitransparent scatter plots were generated using custom MATLAB codes. The shaded boxes in the beeswarm plots indicate mean ± standard error.

## Results

### Biophysical characterization of embryonic stem cells during differentiation

**Fig 2:**
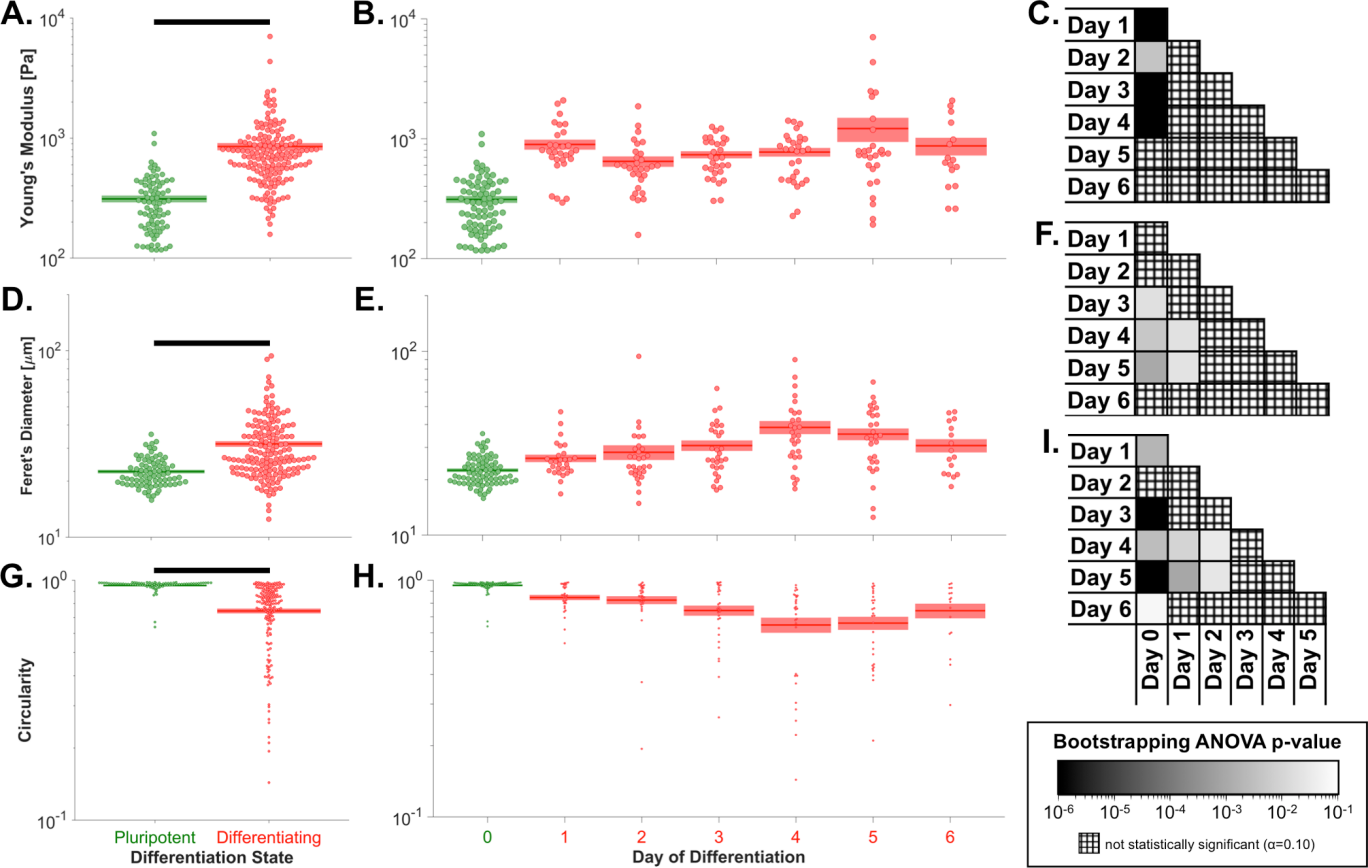
Embryonic stem cells become stiffer, more spread, and less circular during differentiation. (A) Pluripotent ESCs were significantly softer than the pool of all differentiating cells (p*<*10^−6^). (B) Cellular Young’s modulus increased after 1 day of differentiation, and no substantial subsequent change was observed through 6 days of differentiation, indicating the potential utility of Young’s modulus as an early marker of ESC differentiation. (C) Pluripotent cells were significantly softer than cells differentiated for 1–4 days. (D) Pluripotent ESCs had a significantly smaller Feret’s diameter, which indicates spread cell size, than the pool of all differentiating cells (p*<*10^−6^). (E) The Feret’s diameter significantly increased over the first 4 days of differentiation and then decreased by day 6 of differentiation. (F) Pluripotent cells had a significantly lower Feret’s diameter than cells after days 3–5 of differentiation, and day 1 cells had a significantly lower Feret’s diameter than day 4–5 cells. (G) Pluripotent ESCs were significantly more circular than the pool of all differentiating cells (p*<*10^−6^). (H) Circularity significantly decreased over the first 4 days of differentiation and then increased by day 6 of differentiation. (I) Pluripotent cells were significantly more circular than cells differentiated for 1 or 3–6 days, and day 1 and 2 cells were significantly more circular than cells differentiated for 4–5 days. Panels A-B, D-E, & G-H: green, pluripotent cells; red, differentiating cells. Panels A, D, & G: populations connected by black bars are significantly different (α = 0.1). Panels C, F, & I: black, p≤10^−6^; white, p≥10^−1^; cross-hatch, non-significant p-value.

Before addressing the biological properties of ESC biophysical subsets, the characteristics of ESCs at specific days of differentiation were first considered. The cell stiffness values for the full set of paired stiffness-morphology data (N = 359) qualitatively indicated that pluripotent ESCs were softer than differentiated ESCs, with minimal effects of the session (i.e. cantilever spring constant), passage number, differentiation method, and differentiation format used for each sample (S1 Fig). Analysis of cell stiffness for the monolayer, LIF removal differentiation method revealed that ESCs became stiffer during the course of differentiation, with a significant stiffness increase exhibited after only 1 day of differentiation (p_adjusted_<10^−6^, Fig 2A–2C).

The quantification of phase contrast images taken during atomic force microscopy yielded various spread-cell morphological parameters, which were divided into size- and shape-related factors. The Feret's diameter, which represents the longest distance between any two points on the cell border, correlated more strongly with the day of differentiation than any other size factor (ρ = +0.390, p_adjusted_ = 0.009). The circularity, which is defined as $\frac{4∙\pi ∙area}{{perimeter}^{2}}$ and ranges from 0 for an elongated polygon to 1 for a perfect circle, correlated more strongly with the day of differentiation than any other shape factor (ρ = -0.511, p_adjusted_ = 0.009). Analysis of the morphological factors revealed that ESCs became more spread (i.e. increased Feret's diameter) and less circular during differentiation (Fig 2D & 2G); similar trends were qualitatively observed for both ESC colonies and individual ESCs (S2 Fig). Interestingly, the Feret's diameter increased and then decreased during differentiation, whereas the circularity decreased and then increased during differentiation. The extrema of the mean Feret's diameter and circularity both occurred at day 4 (Fig 2E and 2F & 2H and 2I). The inverse relationship between Feret's diameter and circularity may reflect the propensity of ESCs to adopt a more spindle-shaped (i.e. spread, high aspect ratio) morphology during differentiation.

To further understand the observed morphology changes, cells after 0–6 days of differentiation were stained for F-actin and DNA, revealing three distinct morphology types: rounded cells, sheet-like actin, and polarized, fiber-rich actin (S3A Fig). Cells during days 0–1 of differentiation were characterized by the rounded cell morphology, followed by a transition to the sheet-like actin morphology during days 2–5 and finally to the polarized, fiber-rich actin morphology on day 6 (S3B Fig). The observed changes to the actin cytoskeleton align with the observed change from round to spindle-shaped morphology during differentiation.

**Fig 3:**
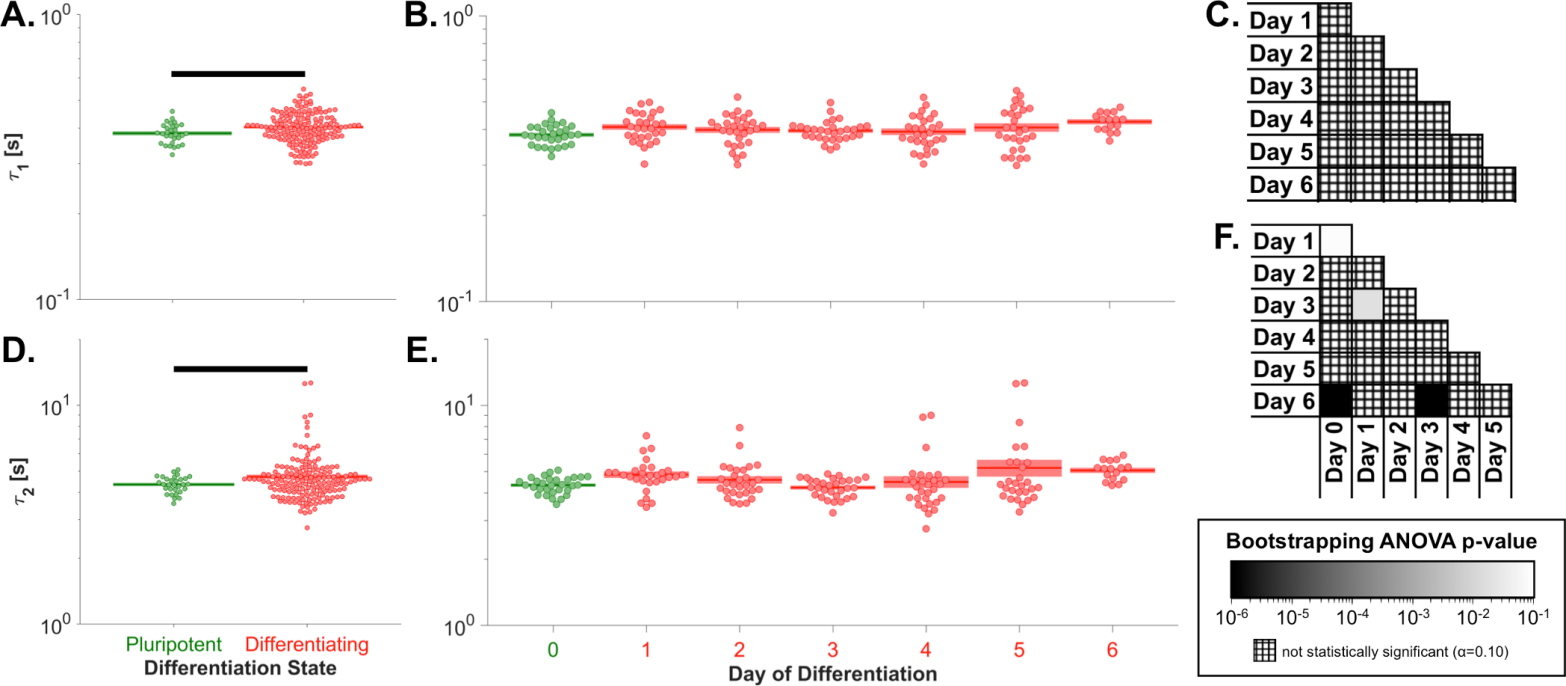
Changes to embryonic stem cell viscoelastic relaxation during differentiation were minimal. (A) Pluripotent ESCs had a significantly lower fast viscoelastic time constant (*τ*_1_) than the pool of all differentiating cells (*p* = 0.005). (B) Changes to the fast viscoelastic time constant were not observed during 6 days of differentiation. (C) The fast viscoelastic time constant was not significantly different between any two days of differentiation (*p*_ANOVA_ = 0.099). (D) Pluripotent ESCs had a significantly lower slow viscoelastic time constant (*τ*_2_) than the pool of all differentiating cells (*p* = 0.007). (E) The slow viscoelastic time constant changed only subtly during 6 days of differentiation. (F) Pluripotent ESCs had a significantly lower slow viscoelastic time constant than day 1 and 6 cells. Significant differences also existed between day 1 and 3 cells and between day 3 and 6 cells. Panels A-B & D-E: green, pluripotent cells; red, differentiating cells. Panels A & D: populations connected by black bars are significantly different (α = 0.1). Panels C & F: black, p≤10^−6^; white, p≥10^−1^; cross-hatch, non-significant p-value.

Analysis of the paired stiffness-viscosity-morphology data (N = 192) revealed that the fast and slow viscoelastic time constants were both lower for pluripotent than differentiated cells; however, significant differences in the time constants between individual days of differentiation were not generally observed (Fig 3). The viscoelastic time constant data are shown in S4 Fig and S5 Fig, with sample letters matched to the data in S1 Fig.

### Sorting pluripotent from differentiating embryonic stem cells

**Fig 4:**
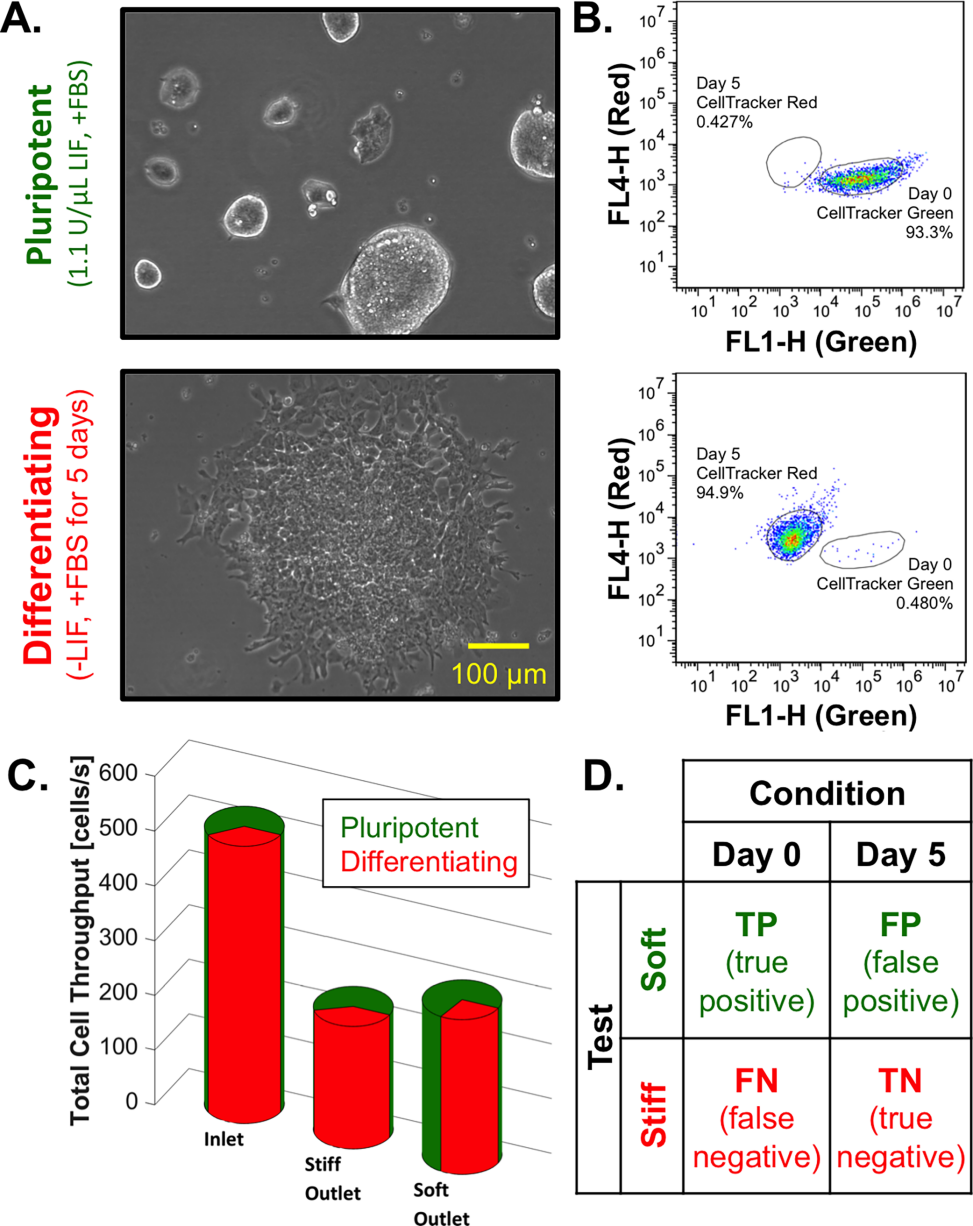
Biophysical separation of day 0 from day 5 embryonic stem cells. (A) Before sorting, the +LIF cell culture displayed pluripotent colonies with rounded morphology and the -LIF cell culture was characterized by differentiated, spindle cell morphology. (B) The pluripotent cells (green) and the differentiating cells (red) displayed distinct fluorescent signatures by cytometry analysis. (C) Starting with a mixture of 67% undifferentiated cells (day 0, green) and 33% differentiating cells (day 5, red), 40% of cells sorted to the stiff outlet were differentiating and 73% of cells sorted to the soft outlet were undifferentiated, indicating relative enrichment in both outlets. (D) To define the sorting efficiency, the contingency table was employed to divide cells by condition (condition positive, day 0; condition negative, day 5) and test (test positive, soft outlet; test negative; stiff outlet). The overall efficiency, defined as the diagnostic odds ratio, was 1.9.

A 2-outlet device with a 15.6 *μ*m gap was employed to sort pluripotent (day 0, +LIF) from differentiating (day 5, -LIF) cells. Cell cultures containing rounded undifferentiated pluripotent colonies or spread differentiating colonies (Fig 4A) were independently stained with CellTracker™ Green and Red, respectively, to distinguish the state of differentiation post-sort (Fig 4B). The cell inlet flow rate was 8 *μ*L/min, and the stiff and soft sheath inlet flow rates were 23 *μ*L/min and 17 *μ*L/min, respectively. The relatively higher stiff sheath inlet flow rate resulted in a slight bias to the soft outlet, which was empirically determined to maximize separation of the day 0 and day 5 cells (Fig 4C).

61% of the undifferentiated (day 0) cells reached the soft outlet and 54% of the differentiating (day 5) cells reached the stiff outlet, serving as a preliminary indicator of pluripotent cell enrichment in the soft outlet. To assess sorting efficiency in more detail, the contingency table was employed to separate sorted cells into true positives (TPs), false positives (FPs), false negatives (FNs), and true negatives (TNs) (Fig 4D; for further information, see [11]). Biophysical sorting was used to select for pluripotent ESCs (condition positive) and against differentiated ESCs (condition negative). Cells sorted to the soft outlet were considered as test positive, and cells sorted to the stiff outlet were considered as test negative.

The efficiency of sorting the day 0 cells, *e*_*day 0*_, was described by the positive likelihood ratio, *LR+*, such that $e_{day\;0}=LR+=\frac{true\;positive\;rate}{false\;positive\;rate}=\frac{\frac{TP}{TP+FN}}{\frac{FP}{FP+TN}}=\frac{\frac{TP}{FP}}{\frac{TP+FN}{FP+TN}}=\frac{{\left(\frac{\%\;day\;0}{\%\;day\;5}\right)}_{soft\;outlet}}{{\left(\frac{\%\;day\;0}{\%\;day\;5}\right)}_{inlet}}$. Similarly, the efficiency of sorting the day 5 cells, *e*_*day 5*_, was described by the multiplicative inverse of the negative likelihood ratio, *LR−*, such that $e_{day\;5}=\frac{1}{LR-}=\frac{true\;negative\;rate}{false\;negative\;rate}=\frac{\frac{TN}{FP+TN}}{\frac{FN}{TP+FN}}=\frac{\frac{TN}{FN}}{\frac{FP+TN}{TP+FN}}=\frac{{\left(\frac{\%\;day\;5}{\%\;day\;0}\right)}_{stiff\;outlet}}{{\left(\frac{\%\;day\;5}{\%\;day\;0}\right)}_{inlet}}$. Thus, the efficiencies of sorting day 0 and 5 cells were 1.6 and 1.2, respectively. The overall sorting efficiency, *e*_*total*_, was described by the diagnostic odds ratio, *DOR*, such that $e_{total}=DOR=\frac{LR+}{LR-}=\frac{\frac{TP}{FP}}{\frac{FN}{TN}}=e_{day\;0}∙e_{day\;5}$. The overall sorting efficiency was 1.9, which is analogous to enriching a mixture from 50% to 62% day 0 cells during a single pass through the device.

### Biophysical subsets

The microfluidic device parameters were first optimized to maximize separation based on cell stiffness. The geometry and sorting parameters of a 2-outlet device determine a single threshold stiffness value that divides the cells simply into “stiff” and “soft” groups. By increasing the number of outlets in the device, the sorting resolution can be improved. A 3-outlet device employs two separate threshold stiffness values, creating the “stiff” and “soft” groups as well as a “middle” group that serves as a buffer. Due to the inclusion of the buffer group, the absolute stiffness difference between cells in the “stiff” and “soft” groups, and therefore the sorting resolution, is greater for a 3-outlet device than a 2-outlet device. Thus, a 3-outlet device containing an additional middle outlet was employed to produce biophysical subsets from a mixed starting cell population.

**Fig 5:**
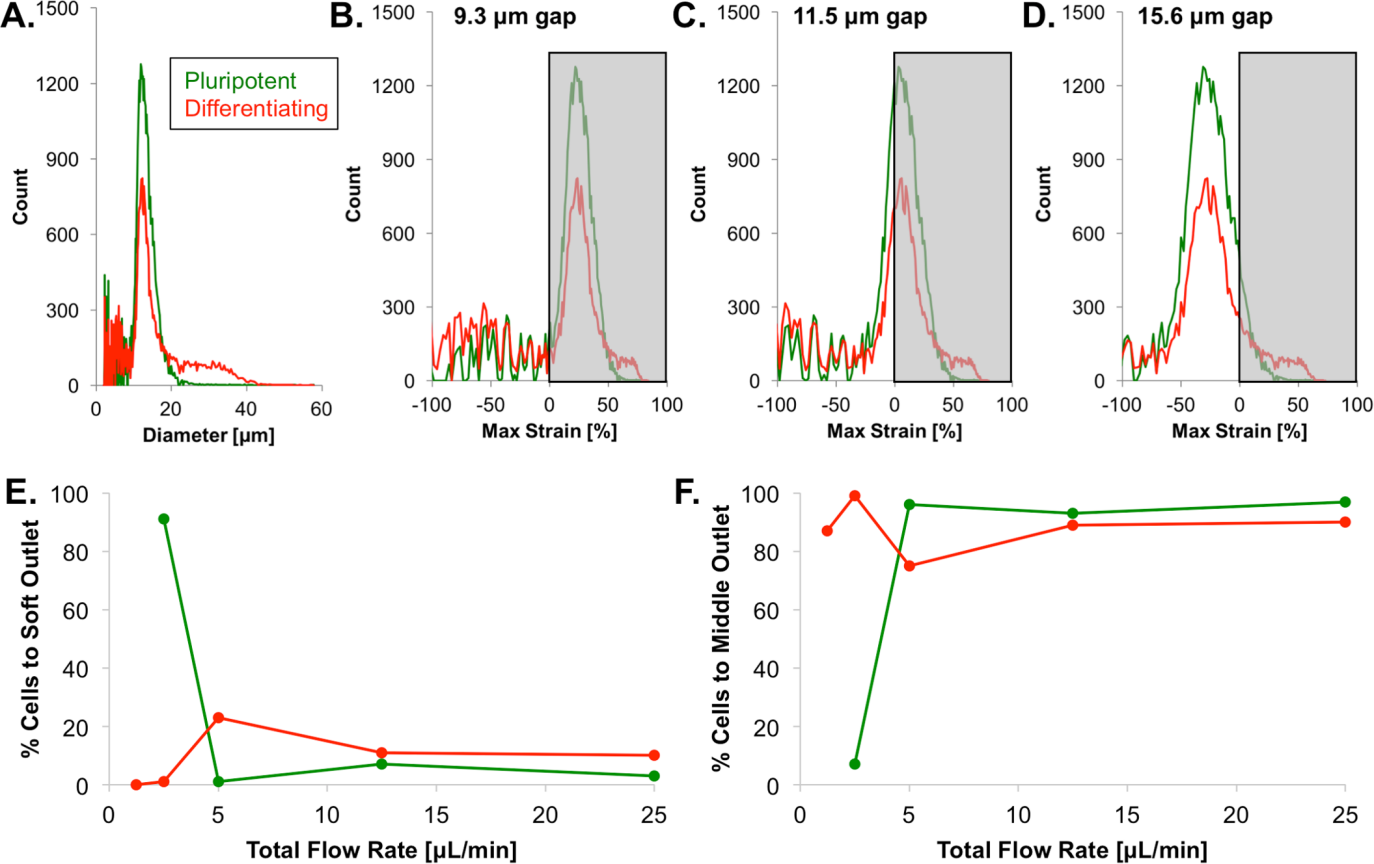
Microfluidic design optimization. (A) The size distribution, which was measured for cells in suspension, was similar for pluripotent (green) and differentiating (red) cells, with modal cell sizes of ~12 *μ*m. (B–D) The design of the microfluidic device requires cells to experience strain for sorting to occur. As the gap size was increased from 9.3 to 15.6 *μ*m, the fraction of cells experiencing strain (gray shading) decreased. However, as the 9.3 *μ*m gap caused device clogging, an 11.5 *μ*m gap was determined to be optimal. (E–F) Optimization studies indicated that a low total flow rate would increase both the fraction of pluripotent cells reaching the soft outlet and the fraction of differentiated cells reaching the middle outlet. The fraction of cells reaching the stiff outlet was negligible compared to the fractions of cells reaching the soft and middle outlets.

The gap size (i.e. the height between the ridge and the glass slide), which affects the strain to which each cell is exposed, was optimized to tune the differential trajectories of pluripotent and differentiated cells. Although the suspended cell size was similar for pluripotent and differentiating cells (Fig 5A), the 15.6 *μ*m gap employed for sorting day 0 from day 5 cells resulted in minimal or no cell strain because the gap size was larger than the average cell size. On the other hand, the 9.3 *μ*m gap caused the device to clog because the gap size was too small relative to the cell size; therefore, a 11.5 *μ*m gap was chosen as an optimal intermediate gap size based on ESC size (Fig 5B–5D).

Although the average viscoelastic properties were relatively unchanged during differentiation, the inter-ridge relaxation time was tuned to reduce viscoelastic-dependent cell separation that could otherwise arise from within-population variations in viscoelasticity, thus favoring cell separation that depended dominantly on stiffness. As the time a cell takes to pass from one ridge to the next depends on both the inter-ridge distance and the overall flow rate, the inter-ridge distance was fixed, and the overall flow rate, which is easier to adjust, was optimized. The lowest flow rates investigated (1.25–2.5 *μ*L/min) maximized the percentage of pluripotent cells reaching the soft outlet and the percentage of differentiating cells reaching the middle outlet (Fig 5E and 5F). Lower flow rates increase the dominance of elastic- over viscous-driven cell separation; thus, the low flow rates maximized the separation of the relatively soft pluripotent cells from the relatively stiff differentiating cells and reduced separation based on the viscoelastic time constants, which were similar for pluripotent and differentiating cells. A total flow rate of 5 *μ*L/min was chosen to both increase the throughput and to reduce the amount of time the cells were exposed to the chemical and thermal environment of the flow buffer, rather than standard cell culture conditions. Both sheath inlet flow rates were set to 2 *μ*L/min, resulting in an unbiased flow profile, and the cell inlet flow rate was set to 1 *μ*L/min.

**Fig 6:**
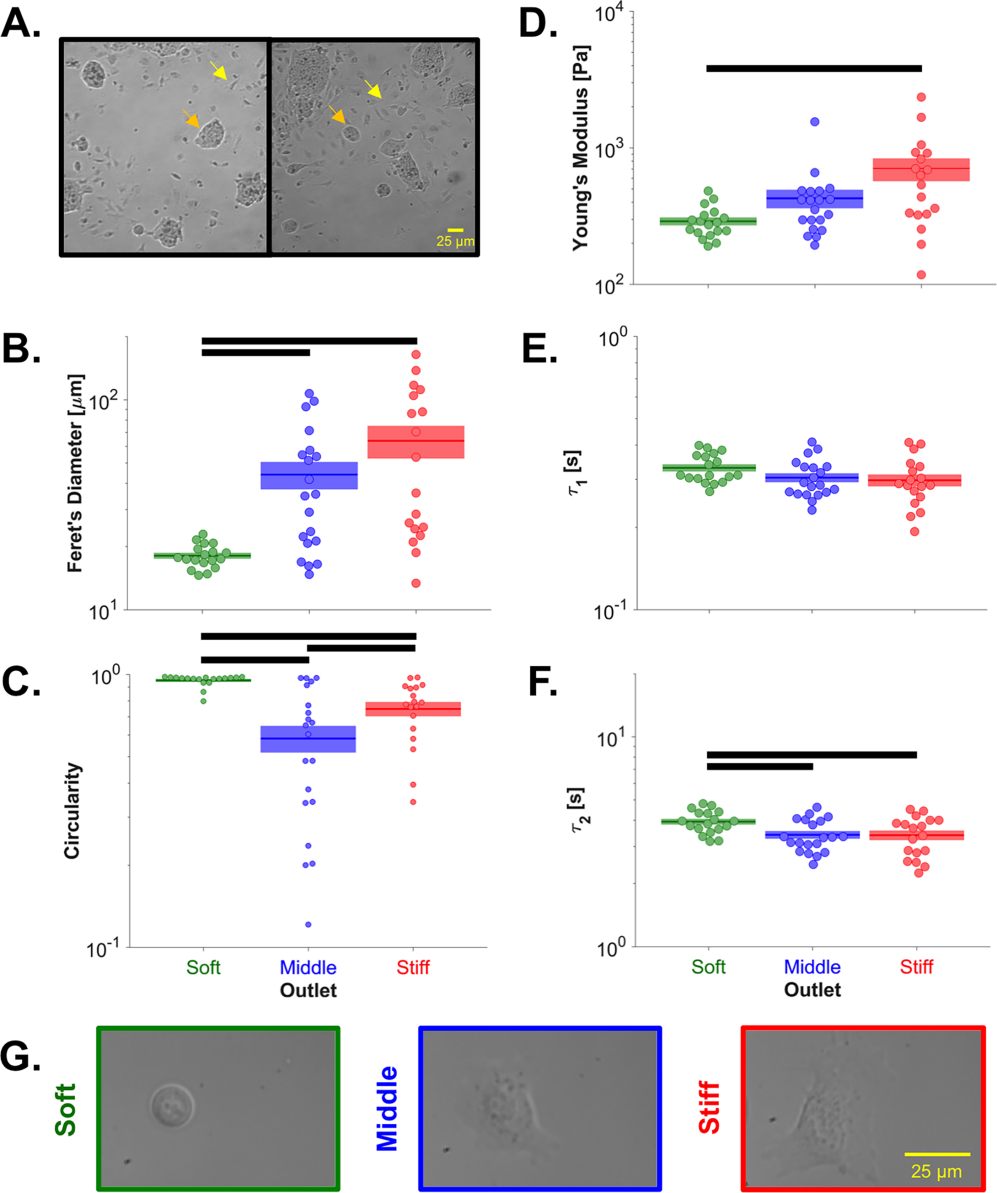
Biophysical characterization after microfluidic stiffness-based sorting. (A) Before sorting, the cell cultures were characterized by a mixture of rounded, pluripotent colonies (e.g. orange arrows) and differentiated cells with spindle-shaped morphology (e.g. yellow arrows). (B) Cells sorted to the soft outlet had a significantly lower Feret’s diameter than cells sorted to the middle (p_adjusted_ = 0.012) or stiff (p_adjusted_ = 0.012) outlet. (C) Cells sorted to the soft outlet were significantly more circular than cells sorted to the middle (p_adjusted_<10^−6^) or stiff (p_adjusted_ = 0.003) outlet. Cells sorted to the stiff outlet were significantly more circular than cells sorted to the middle outlet (p_adjusted_ = 0.042). (D) Cells sorted to the stiff outlet were significantly stiffer than cells sorted to the soft outlet (p_adjusted_ = 0.010). (E) The sorted cells did not have a significantly different fast viscoelastic time constant (p_ANOVA_ = 0.127). (F) Cells sorted to the soft outlet had a significantly higher slow viscoelastic time constant than cells sorted to the middle (p_adjusted_ = 0.019) or stiff (p_adjusted_ = 0.030) outlet. Populations connected by black bars are significantly different (α = 0.1). (G) Representative images of individual cells taken during atomic force microscopy corroborate the quantified Feret’s diameters and circularities.

Biophysical subsets were generated from a cell culture that lacked LIF for 5 days, but contained both pluripotent and differentiating colonies (Fig 6A). Characterization revealed that cells in the soft subset were morphologically similar to the day 0 (+LIF) cells (Fig 2 & S2 Fig), with significantly lower Feret's diameters (Fig 6B & 6G) and higher circularities (Fig 6C & 6G) than cells sorted to the middle and stiff outlets, supporting the conclusion that the soft outlet was enriched for pluripotent cells. Furthermore, cells sorted to the soft outlet were indeed softer (by ~60%) than cells sorted to the stiff outlet (Fig 6D). The fast viscoelastic time constant was not different between the biophysical subsets (Fig 6E), as observed during ESC differentiation. The increased slow time constant in the soft biophysical subset (Fig 6F) did not indicate increased pluripotency in the soft biophysical subset but may reflect the mechanism of cell sorting, which causes both soft and more viscous cells to be sorted to the soft outlet.

### Gene target selection

The expression of housekeeping genes (*Gapdh*, *Rps18*), pluripotency genes (*Nanog*, *Pou5f1*, *Sox2*), differentiation genes (*Isl1*, *Map2*), and structural genes (*Actn1*, *Lmna*, *Map2*) was investigated both over the course of differentiation and for the soft, middle, and stiff biophysical subsets. *Nanog*, *Pou5f1* (*Oct-4*), and *Sox2* are common markers of ESC pluripotency that are important to ESC self-renewal [26–30]. *Isl1* (islet-1), which can indicate differentiation to any of the three germ lineages, including spinal motor neurons, pan-endocrine tissues, and cardiomyocytes [31–33], was employed as a general marker of ESC differentiation. *Actn1* (α-actinin-1) crosslinks and bundles F-actin filaments, increases the stiffness of the actin filament network, and is known to increase as ESCs differentiate to cardiac progenitor cells [12,34,35]. Expression of *Lmna* (lamin A/C) is associated with stiff nuclei [36] and differentiated mouse embryonic tissue [10,37,38]. *Map2* (microtubule-associated protein 2) expression is associated with both ectoderm differentiation and microtubule assembly [39,40].

### Gene expression of embryonic stem cells by day of differentiation

**Fig 7:**
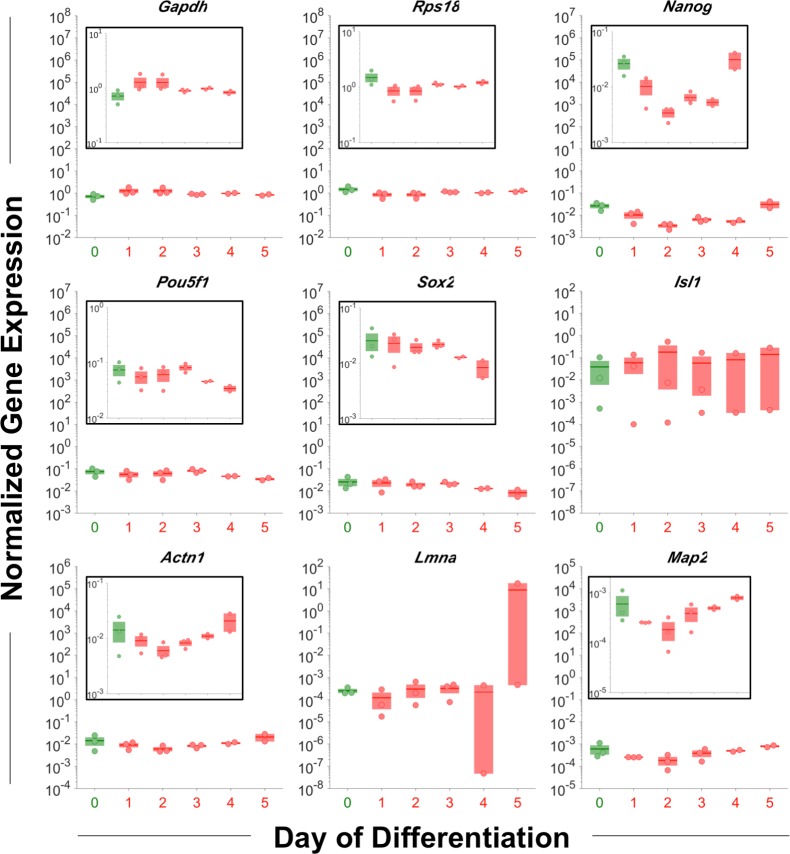
Gene expression by day of differentiation. Over 5 days of differentiation, the pluripotency genes generally decreased, with changes to *Pou5f1* and *Sox2* lagging changes to *Nanog*. As for the structural genes, *Actn1* and *Map2* both decreased and then increased, and *Lmna* was stable until a sharp increase on day 5. N_0_ gene expression values were normalized by the geometric mean of the housekeeping genes *Gapdh* and *Rps18*. Green, pluripotent cells; red, differentiating cells. No significant differences were observed among the days of differentiation for any gene considered (pairwise t-tests, log-transformed values, α = 0.05).

Gene expression trends during differentiation were assessed via *N*_*0*_ gene expression values that were normalized by the geometric mean of the housekeeping genes *Gapdh* and *Rps18*, as previously described [41]. Over the course of differentiation, a dynamic gene expression profile was observed for most genes. The pluripotency genes generally decreased, with *Nanog* beginning to change on day 1 and *Pou5f1* and *Sox2* lagging with changes beginning on day 4 (Fig 7), which is supported by previous reports that *Nanog* regulates the expression of *Pou5f1* and *Sox2* [42,43]. As *Isl1* is not typically detected until days 4–8 of differentiation [44,45], 5 days of differentiation may have been insufficient for detection. *Actn1* and *Map2* both decreased and then increased (Fig 7), which reflect that expression is typically delayed until day 5–18 or 6–16 of differentiation, respectively [34,46–50]. *Lmna* was characterized by minimal changes until a sharp increase on day 5 (Fig 7), which is consistent with previous reports that *Lmna* is not expressed until the mid-to-late state of differentiation [51–53].

### Gene expression of embryonic stem cells by biophysical subset

ESCs were partially differentiated before being sorted into biophysical subsets for assessment of gene expression. For sorting experiments #1–3, ESCs were differentiated for 3 days, at which point they contained both pluripotent- and differentiated-morphology colonies; sorting experiment #4 employed a mixture of day 0 and day 3 cells (S6 Fig).

Initially, the 100-cell samples collected from sorting experiments #1–3 (n = 1 100-cell sample) and sorting experiment #4 (n = 3 100-cell samples) were combined as a single set with n = 6, as the between-experiment and between-replicate gene expression variabilities were similar (S7 Fig). Gene expression fold-changes were calculated using the ΔΔ*C*_t_ method, with the housekeeping gene as the geometric mean of *Gapdh* and *Rps18*. Increased *Sox2* expression in the soft outlet (S8 Fig) suggests the enrichment of pluripotent ESCs in the soft outlet, as predicted from the day of differentiation (Fig 2A–2C) and post-sort (Fig 6D) stiffness data. Increased *Actn1* in the middle and stiff outlets (S8 Fig) indicates that F-actin crosslinking and bundling may contribute to ESC stiffness. However, only subtle gene expression changes were observed for ΔΔ*C*_t_ analysis of the combined n = 6 data set (S8 Fig).

**Fig 8:**
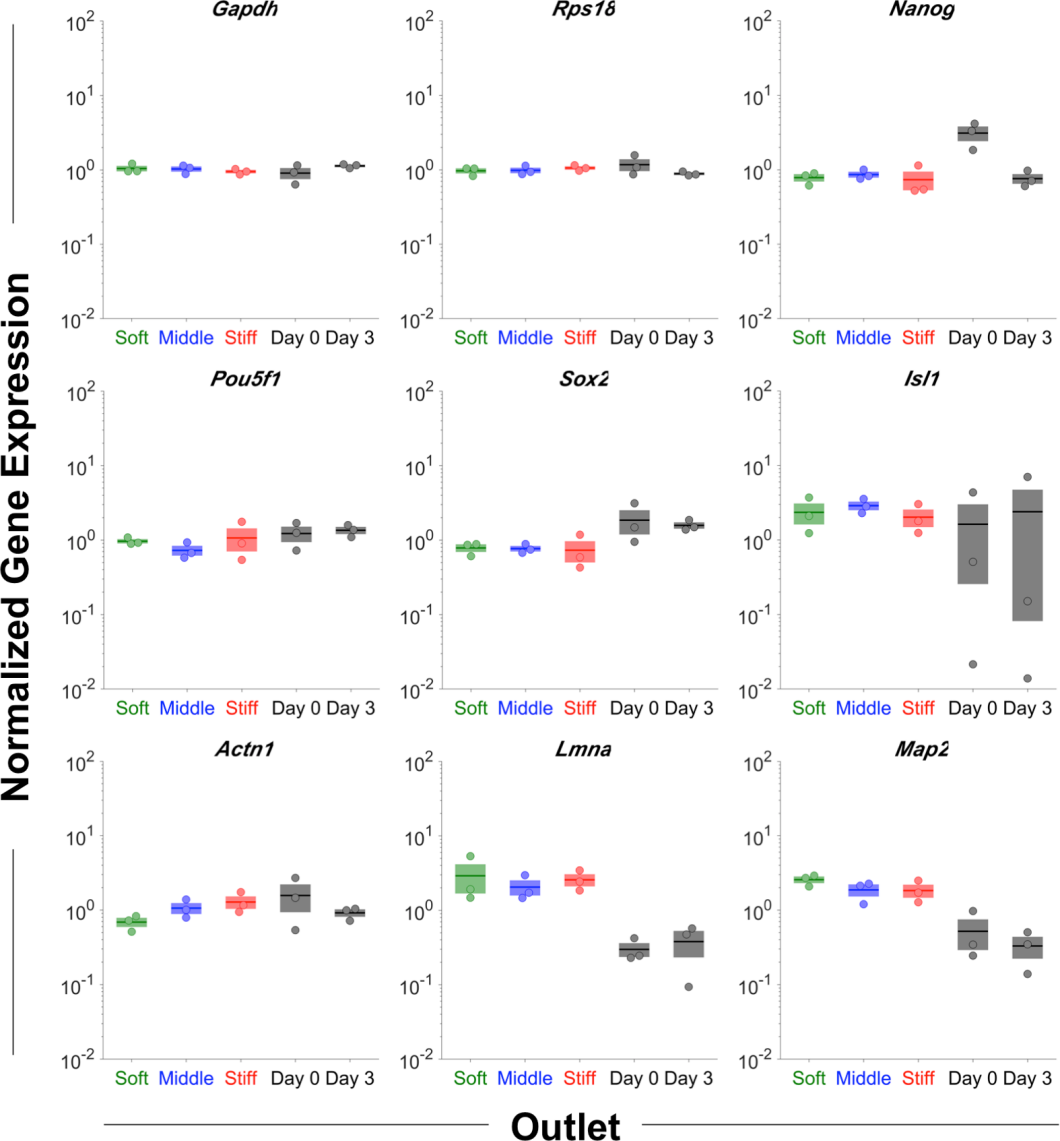
Gene expression by biophysical subset. An enrichment of pluripotent cells in the soft outlet was indicated by decreased *Nanog* in the stiff outlet and increased *Pou5f1* in the soft outlet, relative to the middle outlet. The monotonic increase in *Actn1* from the soft to middle to stiff outlet implicates F-actin crosslinking in the stiffness differences observed between the biophysical subsets. N_0_ gene expression values were normalized by the geometric mean of the housekeeping genes *Gapdh* and *Rps18*. Day 0 and Day 3 indicate unsorted control cells. No significant differences were observed among the 3 outlets for any gene considered (pairwise t-tests, log-transformed values, α = 0.05).

Therefore, to resolve trends in gene expression, further analysis was constrained to sorting experiment #4, which employed a mixture of day 0 and day 3 cells to maximize the range of biological signals. Relative to the middle outlet, the pluripotency gene *Nanog* was decreased in the stiff outlet and the pluripotency gene *Pou5f1* was increased in the soft outlet (Fig 8), indicating the enrichment of pluripotent cells in the soft outlet. The expression of *Actn1* continuously increased from the soft to middle to stiff outlet (Fig 8), indicating that F-actin crosslinking may play a role in stiffness differences between the biophysical subsets, which is corroborated by evidence that *Actn1* is responsible for stiffening both actin filament networks and entire cells [54,55]. Taken together, the changes to the pluripotency and structural genes indicate the relative enrichment of soft, pluripotent cells in the soft device outlet.

In addition to sorting cells based on potency, the microfluidic device presented herein has potential applications to select for specific differentiated cell phenotypes, rescue an over-confluent ESC culture, or remove the feeder layer from an ESC culture in a label-free, high-throughput manner. To this end, pluripotent ESCs and mouse embryonic fibroblasts (MEFs) were employed as a model system for sorting. Although human pluripotent stem cells and fibroblasts are known to differ in adhesion strength [56], a significant stiffness difference was not observed between ESCs and MEFs (S9A Fig). However, size-based sorting has been noted in the microfluidic sorting literature [57]. For the device presented herein, when differences in cell stiffness are minimal, the microfluidic device can sort cells based on size via a similar physical principle, with small cells reaching the soft outlet and large cells reaching the stiff outlet. Thus, the size difference between the two cell types (S9B and S9C Fig) was exploited to drive microfluidic separation. Sorting using a device with a 15.6 *μ*m gap size resulted in a ESC sorting efficiency of 3.4 and a MEF sorting efficiency of 3.3 (S9D Fig); thus, the overall sorting efficiency, *e*_*total*_, was 11.2.

## Discussion

Characterizing ESCs with known days of differentiation revealed that the cells stiffen within 1 day of differentiation and, on average, remain at a similar stiffness level for at least 5 more days, while changes to the viscoelastic relaxation response of cells were minimal. An increase in Feret’s diameter and a concomitant decrease in circularity were also observed as differentiation progressed. After sorting cells by stiffness using a microfluidic device, pluripotent cells were enriched in the soft outlet and differentiated cells were enriched in the stiff outlet. Using a 3-outlet device to sort a mixed population of pluripotent and differentiated cells, the soft subset of cells was more characteristic of the known day 0 cell population than the middle or stiff subsets, as assessed by stiffness and morphology. An assessment of the gene expression levels of sorted cells revealed decreased *Nanog* in the stiff outlet, increased *Pou5f1* in the soft outlet, and increased *Actn1* in the middle and stiff outlets, which reflect the enrichment of pluripotent cells in the soft outlet of the device.

In agreement with the present study, ESCs have previously been observed to stiffen after differentiation both as single cells [10,38] and intact embryoid bodies [14], but the present study is the first to observe that stiffness changes may precede morphology changes. As pluripotent stem cells are known to have a different cytoskeletal structure than more differentiated cells [58] and the rearrangement of the actin cytoskeleton is known to precede the loss of the pluripotency gene *Pou5f1* [59], changes to the cell structure during differentiation may underpin the observed stiffening effect. The dynamic changes in Feret’s diameter and circularity (Figs 2E & 6H) are closely related to changes in *Actn1* (Fig 7), with a 2-day lag that may indicate the expected delay of functional changes behind gene expression changes. The apparent connection between cellular spread area, roundness, and *Actn1* expression reflects a previous finding that *Actn1* controls cellular shape plasticity and the reaction to mechanical cues [55]. Furthermore, pluripotent ESCs have reduced levels of lamin A/C, resulting in an open chromatin state and irregular nuclear shape [37] that is linked to decreased cell stiffness [36]. Previous reports have also suggested a role of chromatin condensation in ESC stiffening during differentiation [10].

**Fig 9:**
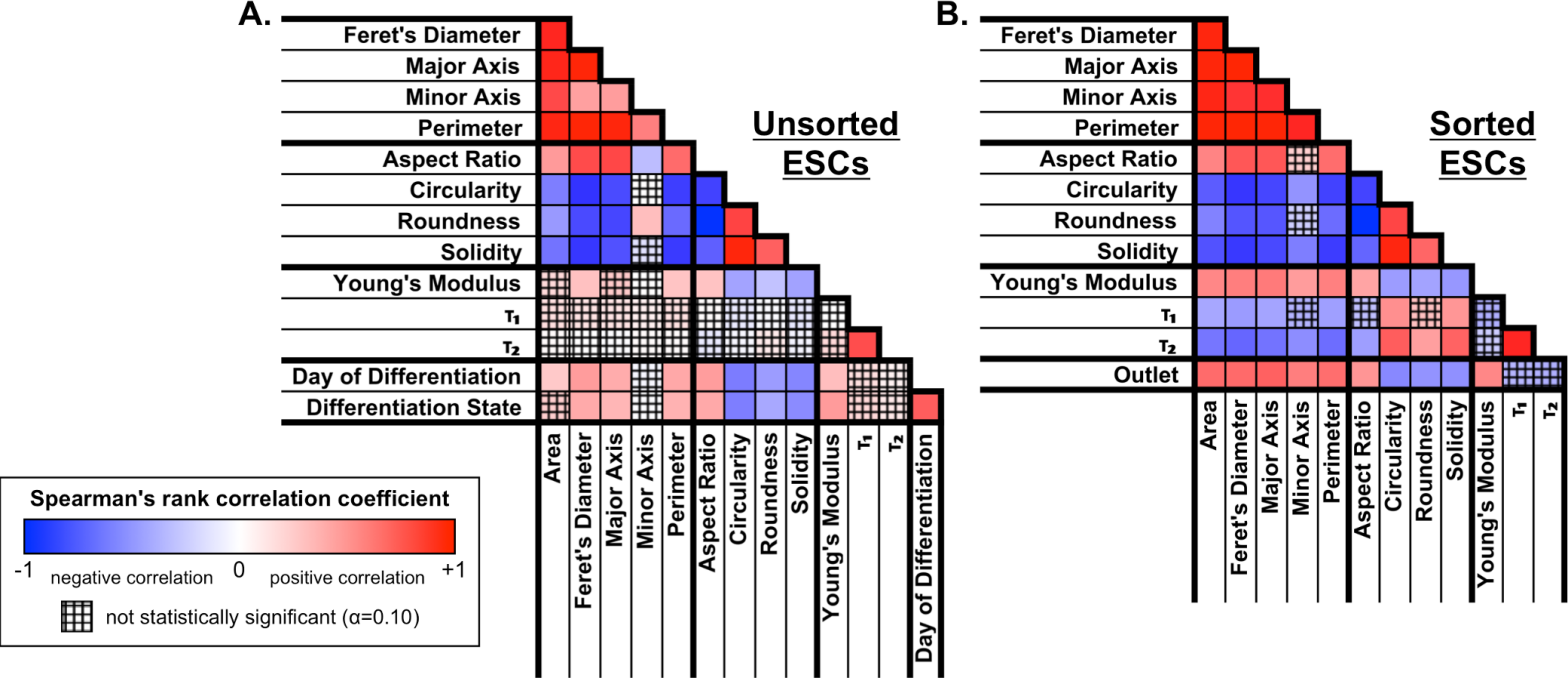
Biophysical correlation color maps for unsorted and sorted ESCs. (A) Spearman's correlations, which indicate monotonic trends for pairs of biophysical parameters, were compared for unsorted ESCs with known day of differentiation and differentiation state (0, pluripotent; 1, differentiating). Differentiation was positively correlated with spread cell size (high area, Feret's diameter, major axis, perimeter) and stiffness (high Young's modulus), but negatively correlated with spread cell roundness (high aspect ratio; low circularity, roundness, solidity). (B) Spearman's correlations were also calculated following microfluidic sorting to generate biophysical subsets, i.e. based on outlet (-1, soft outlet; 0, middle outlet; 1, stiff outlet). The stiff outlet tended to have cells that were more spread (high area, Feret's diameter, major axis, minor axis, perimeter), more spindle-shaped (high aspect ratio; low circularity, roundness, solidity), and stiffer (high Young's modulus). Overall, the correlations between pairs of biophysical parameters were similar for unsorted ESCs relative to the day of differentiation or differentiation state (A) and for sorted ESCs relative to the microfluidic outlet (B). Blue, negative Spearman's correlation coefficient (indirect relationship); red, positive Spearman's correlation coefficient (direct relationship); white, zero Spearman's correlation coefficient (no correlation); cross-hatch, non-significant p-value.

**Fig 10:**
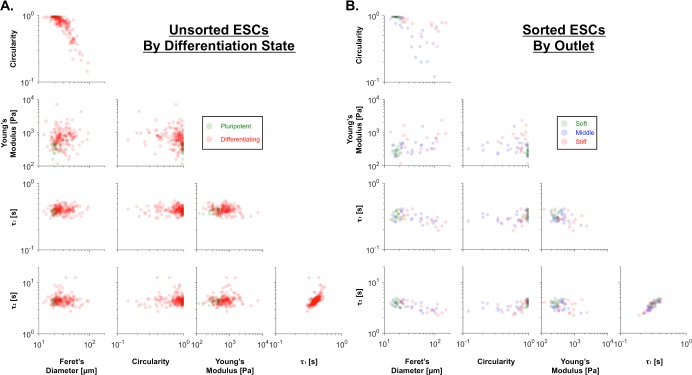
Similarities between pairwise biophysical signatures of pluripotent cells and the soft biophysical subset. (A) Compared to the differentiating cells (red), the pluripotent cells (green) were softer, less spread, and more circular. (B) The biophysical characteristics were similar for cells sorted to the soft outlet (green) and pluripotent cells, whereas cells sorted to the middle (blue) and stiff (red) outlets resembled differentiating cells.

To further assess the relationship between differentiation and the sorted biophysical subsets, Spearman's correlation coefficients were calculated for each pair of parameters, taking into account either cells with a known day of differentiation (Fig 9A) or cells sorted into biophysical subsets (Fig 9B). The correlation coefficients indicated that both pluripotent cells and cells sorted to the soft outlet were soft, less spread, and circular (Figs 9 & 10), supporting the conclusion that the microfluidic device successfully enriched for pluripotent cells in the soft outlet.

Importantly, the trends observed for the unsorted ESCs held regardless of data set expansion to include cells lacking viscoelastic relaxation data (S10 Fig), indicating that the results were not artifacts of the particular data subset chosen for analysis. Strikingly, Young's modulus was the only parameter that correlated more strongly with differentiation state (i.e. pluripotent vs. differentiating) than the day of differentiation for both stiffness and stiffness-viscoelasticity data, indicating that Young's modulus may serve as a better binary potency classifier than the other parameters measured in this study. The differences in day of differentiation vs. spread cell size trends between the original and expanded data sets (S10 Fig) can be explained by the substantial size difference between the monolayer- and embryoid body-derived cells; on average, relative to the monolayer-derived cells, the embryoid body-derived cells were 35% smaller based on Feret’s diameter and 48% smaller based on spread area.

Young’s modulus was also used as a binary classifier in a previous study that established stiffness-based sorting of ESCs [60]. Tangential flow filtration was employed to separate ESCs from ESC-derived osteoblasts or fibroblasts based on Young's modulus. Interestingly, the Young's modulus increased, decreased, then increased again during osteoblast differentiation. Despite the lack of a monotonic trend, stiffness separation between ESCs and osteoblasts was achieved for all days of differentiation considered. Aside from the flow cytometry markers used to assess sorting efficiency, the previous study did not include any post-sort phenotyping, such as the mechanical, morphological, and gene expression characterization employed in the present study.

Further experiments will be required to improve understanding of the interplay between cell structure and mechanics and to explore additional applications of stemness sorting based on cell biophysics. Since cell nuclei are generally observed to be stiffer than the cytoskeleton [61] and ESC nuclei occupy a large volume of the cell [10], the ESC stiffness would be expected to change along with nuclear stiffness. Further exploration of this relationship could be completed by isolating cell nuclei and comparing overall cell stiffness to nuclear stiffness. Whereas the relationship between cell mechanics and cytoskeletal structure is well understood in adherent, spread cells, such as MSCs, the relationship is not understood as well in columnar, epithelial-like cells, such as ESCs. Investigation of cell structure, based on either the day of differentiation or the biophysical subset, could be achieved by staining cells for nuclear material and cytoskeletal components.

## Conclusions

In the present study, pluripotent ESCs were enriched via mechanically-driven cell sorting, which highlights cell mechanics as a basis for efficient, high-throughput isolation of pluripotent ESCs. Further optimization of cell sorting parameters, such as flow rate, cell concentration, and device geometry, in addition to employing multiple sorts in series, will enable stiffness-based, microfluidic sorting to be used as a novel, label-free, and highly efficient method for the purification of pluripotent ESCs. The ability to generate pure populations of pluripotent ESCs will facilitate a greater understanding of pluripotency and serve as a step toward realizing the potential of ESCs as cell sources for various applications. Technologies that can select for or against pluripotent cells, such as stiffness-based microfluidic sorting, also hold great potential to be adapted for the enrichment of specific differentiated lineages, with applications to improving directed differentiation for regenerative medicine and tissue engineering.

## Supporting information

S1 File biophysical characterization_mEF_pre-sort.xlsx. Biophysical Characterization—MEF, Pre-Sort. Cellular mechanics measurements obtained via atomic force microscopy for mouse embryonic fibroblasts prior to microfluidic sorting. **biophysical characterization_mESC_post-sort.xlsx.** Biophysical Characterization—mESC, Post-Sort. Cellular mechanics measurements obtained via atomic force microscopy and morphology measurements obtained via ImageJ for mouse embryonic stem cells following microfluidic sorting. **biophysical characterization_mESC_pre-sort.xlsx.** Biophysical Characterization—mESC, Pre-Sort. Cellular mechanics measurements obtained via atomic force microscopy and morphology measurements obtained via ImageJ for mouse embryonic stem cells prior to microfluidic sorting. **gene expression.xlsx.** Pre- and Post-Sort Gene Expression Data. Single-cell PCR data obtained for mouse embryonic stem cells before or after microfluidic sorting. **size_pluripotent mESC-differentiating mESC-mEF.xlsx.** Suspended Cell Size Data. Size data for pluripotent mouse embryonic stem cells, differentiating mouse embryonic stem cells, and mouse embryonic fibroblasts obtained via a Coulter Counter. **sort_mESC-mEF_inlet.fcs.** Flow Cytometry Data—Sorting of ESCs and MEFs—Inlet. FL1, ESC; FL4, MEF. **sort_mESC-mEF_mEF control.fcs.** Flow Cytometry Data—MEF Control. FL1, ESC; FL4, MEF. **sort_mESC-mEF_mESC control.fcs.** Flow Cytometry Data—ESC Control. FL1, ESC; FL4, MEF. **sort_mESC-mEF_soft outlet.fcs.** Flow Cytometry Data—Sorting of ESCs and MEFs—Soft Outlet. FL1, ESC; FL4, MEF. **sort_mESC-mEF_stiff outlet.fcs.** Flow Cytometry Data—Sorting of ESCs and MEFs—Stiff Outlet. FL1, ESC; FL4, MEF. **sort_pluripotent mESC-differentiating mESC_differentiating control.fcs.** Flow Cytometry Data—Differentiating ESC Control. FL1, pluripotent; FL4, differentiating. **sort_pluripotent mESC-differentiating mESC_inlet.fcs.** Flow Cytometry Data—Sorting of pluripotent and differentiating ESCs—Inlet. FL1, pluripotent; FL4, differentiating. **sort_pluripotent mESC-differentiating mESC_pluripotent control.fcs.** Flow Cytometry Data—Pluripotent ESC Control. FL1, pluripotent; FL4, differentiating. **sort_pluripotent mESC-differentiating mESC_soft outlet.fcs.** Flow Cytometry Data—Sorting of pluripotent and differentiating ESCs—Soft Outlet. FL1, pluripotent; FL4, differentiating. **sort_pluripotent mESC-differentiating mESC_stiff outlet.fcs.** Flow Cytometry Data—Sorting of pluripotent and differentiating ESCs—Stiff Outlet. FL1, pluripotent; FL4, differentiating.
(ZIP)

S1 Fig Young's modulus depends more on differentiation state than other factors. Among the 13 samples probed during 4 atomic force microscopy sessions, effects of the day 0 passage number, the differentiation method, and the differentiation format were dominated by the effect of the differentiation state, i.e. pluripotent (green) vs. differentiating (red). LIF, leukemia inhibitory factor; FBS, fetal bovine serum; BMP-4, bone morphogenetic protein 4; ESGRO, ESGRO complete basal medium (Millipore); mono, monolayer; EB, embryoid body.
(TIF)

S2 Fig ESC Morphology changes during differentiation. Over 6 days of differentiation, images of ESC populations depicted a transition from smaller, rounded colonies to larger, spread colonies (top row). Similarly, individual cells, which were mechanically characterized by atomic force microscopy, became more spread and less circular during differentiation (bottom 3 rows). For each day of differentiation, the single-cell images represent the cell with the upper quartile, median, and lower quartile value of Feret’s diameter.
(TIF)

S3 Fig Cytoskeletal remodeling during differentiation. (A) Cells were stained for F-actin (fluorescent green) using phalloidin and for DNA (fluorescent blue) using Hoescht 33342. Cell morphologies were categorized as one of three types: rounded cells (left), sheet-like actin (middle), or polarized, fiber-rich actin (right). (B). As shown in the doughnut plots, the dominant morphology type changed from rounded cells (green) on days 0–1 to sheet-like actin (blue) on days 2–5 and finally to polarized, fiber-rich actin (red) on day 6. Representative images were selected from the majority morphological type for each day of differentiation. Scale bars indicate 10 μm.
(TIF)

S4 Fig The fast viscoelastic time constant, *τ*_1_, was independent of the day of differentiation. The sample letters are matched to the data in S1 Fig. For all cells, the day 0 session was 4, the day 0 passage number was 28, and differentiation was induced via leukemia inhibitory factor removal in the presence of fetal bovine serum in monolayer format.
(TIF)

S5 Fig The slow viscoelastic time constant, *τ*_2_, was independent of the day of differentiation. The sample letters are matched to the samples in S1 Fig. For all cells, the day 0 session was 4, the day 0 passage number was 28, and differentiation was induced via leukemia inhibitory factor removal in the presence of fetal bovine serum in monolayer format.
(TIF)

S6 Fig Pre-sort colony morphology. Before microfluidic sorting, the ESC cultures contained both rounded pluripotent colonies and spread differentiating colonies. For sorts 1–3, “pluripotent” and “differentiated” indicate colonies with the respective morphologies after 3 days of differentiation. For sort 4, day 0 cells with pluripotent morphology and day 3 cells with differentiated morphology were mixed prior to sorting.
(TIF)

S7 Fig Combination of biophysical subset gene expression replicates. Following biophysical separation, 100-cell samples were collected for gene expression analysis. For the first 3 separation experiments (blue, red, and green circles), n = 1 100-cell replicate was collected per outlet. For the fourth separation experiment (black triangles), n = 3 100-cell replicates were collected per outlet. As the between-experiment and between-replicate initial target DNA z-scores was not substantially different, initial analysis was conducted using the pooled set of n = 6 100-cell samples (see S8 Fig).
(TIF)

S8 Fig Gene expression by biophysical subset, initial analysis. The pluripotency gene *Sox2* was increased in the soft outlet, although *Nanog* and *Pou5f1* showed unclear trends. The structural gene *Actn1* increased in the middle and stiff outlets. Green, soft outlet; blue, middle outlet; red, stiff outlet; ΔΔ*C*_t_ values, control group = soft outlet, housekeeping gene = geometric mean of *Gapdh* and *Rps18*; mean of six 100-cell samples (see S7 Fig).
(TIF)

S9 Fig Biophysical separation of embryonic stem cells from mouse embryonic fibroblasts. (A) There was no significant difference between the stiffness of mouse embryonic stem cells (ESCs, green) and mouse embryonic fibroblasts (MEFs, red; p = 0.329). (B) However, the cell diameter, which was measured for cells in suspension, was generally smaller for ESCs than MEFs and thus represented an independent biophysical parameter that is suitable for microfluidic sorting. (C) A 15.6 *μ*m gap size was chosen to expose the majority of ESCs and MEFs to strain (gray shading), maximizing the differential sorting trajectory between cell types. (D) The sorting efficiencies of ESCs in the soft (small) outlet and MEFs in the stiff (large) outlet, defined similarly to *e*_day 0_ and *e*_day 5_, respectively, both exceeded 3.
(TIF)

S10 Fig Trends between pairs of biophysical parameters did not change substantially for expanded data sets. Compared to Fig 9A, similar relationships among spread cell area, spread cell roundness, mechanics, and differentiation were observed when the data set was expanded to include cells for which viscoelastic data were not available. The first data expansion included only cells differentiated in monolayer by LIF removal (A, N = 242), and the second data expansion considered all cells, including cells differentiated in embryoid body format (B, N = 359). As viscoelastic relaxation profiles were not recorded for large portions (21% and 47%, respectively) of cells in the expanded data sets, relationships with the viscoelastic relaxation time constants were not considered.
(TIF)

S1 Table Primer sequences. Primers employed for pre-amplification and PCR.
(DOCX)
