# Supplementary figures and images for: Biophysical subsets of embryonic stem cells display distinct phenotypic and morphological signatures
Source: PLoS One. 2018 Mar 8;13(3):e0192631. doi: 10.1371/journal.pone.0192631 (PMC5843178; doi:10.1371/journal.pone.0192631)

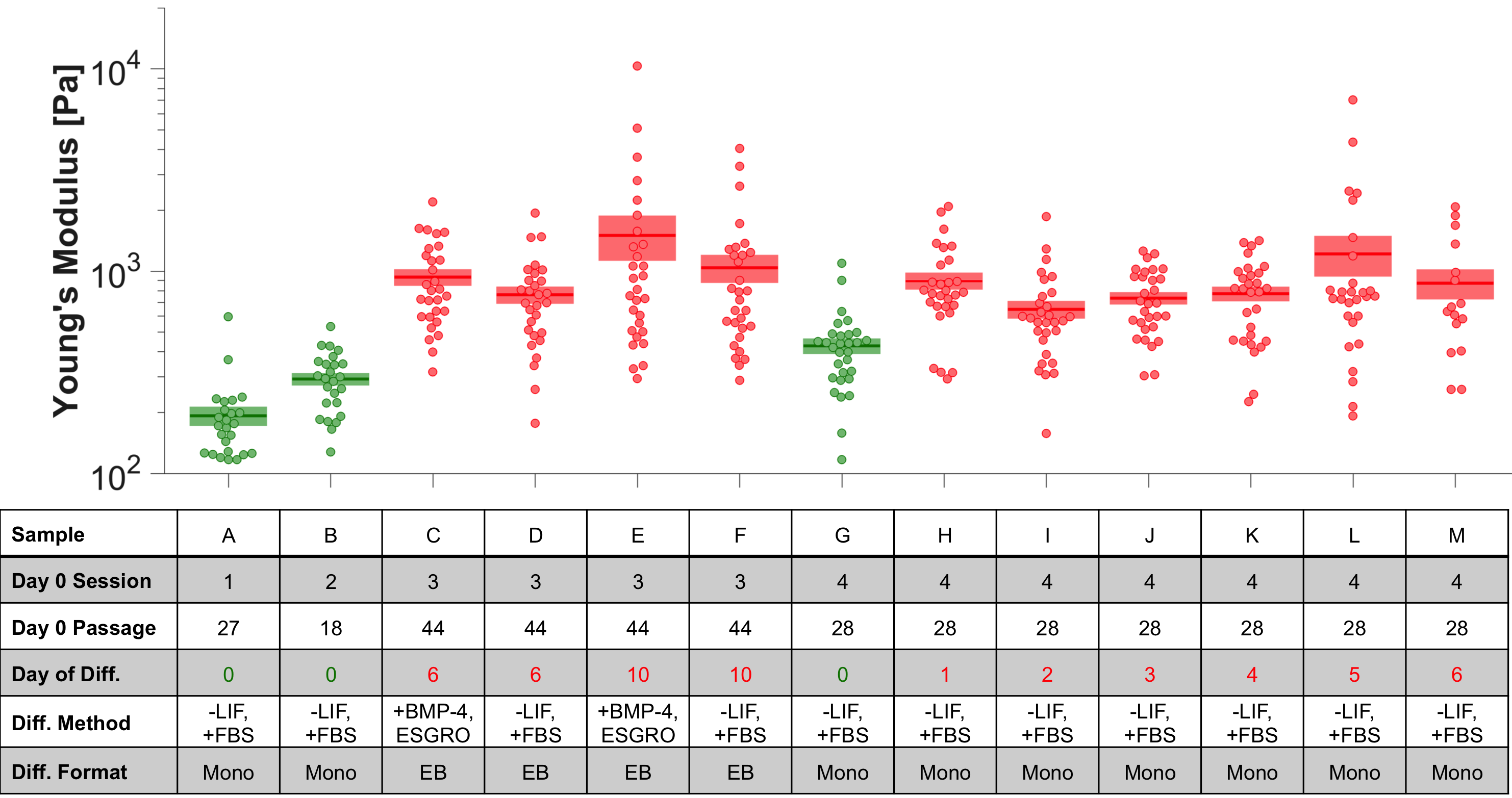

Supplement: S1 Fig — Among the 13 samples probed during 4 atomic force microscopy sessions, effects of the day 0 passage number, the differentiation method, and the differentiation format were dominated by the effect of the differentiation state, i.e. pluripotent (green) vs. differentiating (red). LIF, leukemia inhibitory factor; FBS, fetal bovine serum; BMP-4, bone morphogenetic protein 4; ESGRO, ESGRO complete basal medium (Millipore); mono, monolayer; EB, embryoid body. (TIF) [file pone.0192631.s002.tif]

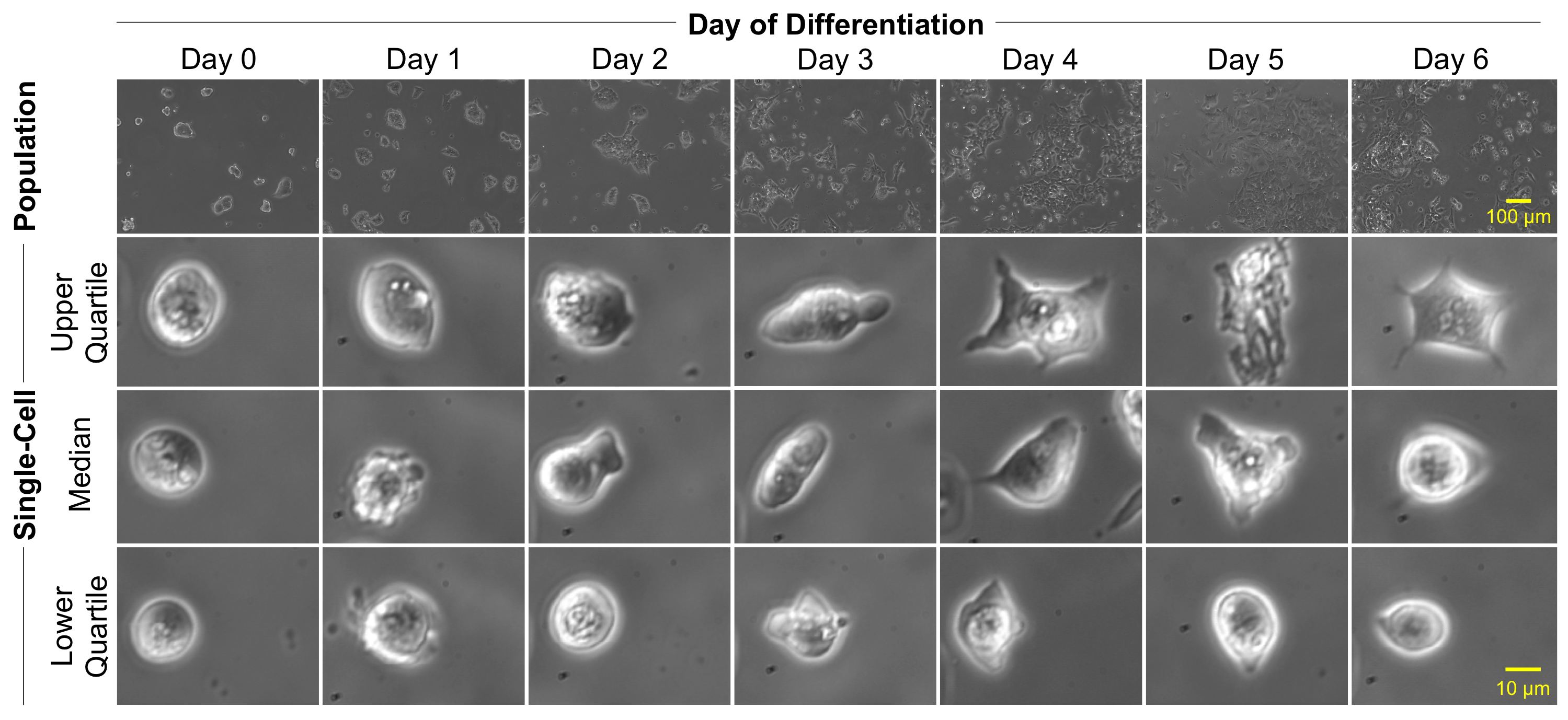

Supplement: S2 Fig — Over 6 days of differentiation, images of ESC populations depicted a transition from smaller, rounded colonies to larger, spread colonies (top row). Similarly, individual cells, which were mechanically characterized by atomic force microscopy, became more spread and less circular during differentiation (bottom 3 rows). For each day of differentiation, the single-cell images represent the cell with the upper quartile, median, and lower quartile value of Feret’s diameter. (TIF) [file pone.0192631.s003.tif]

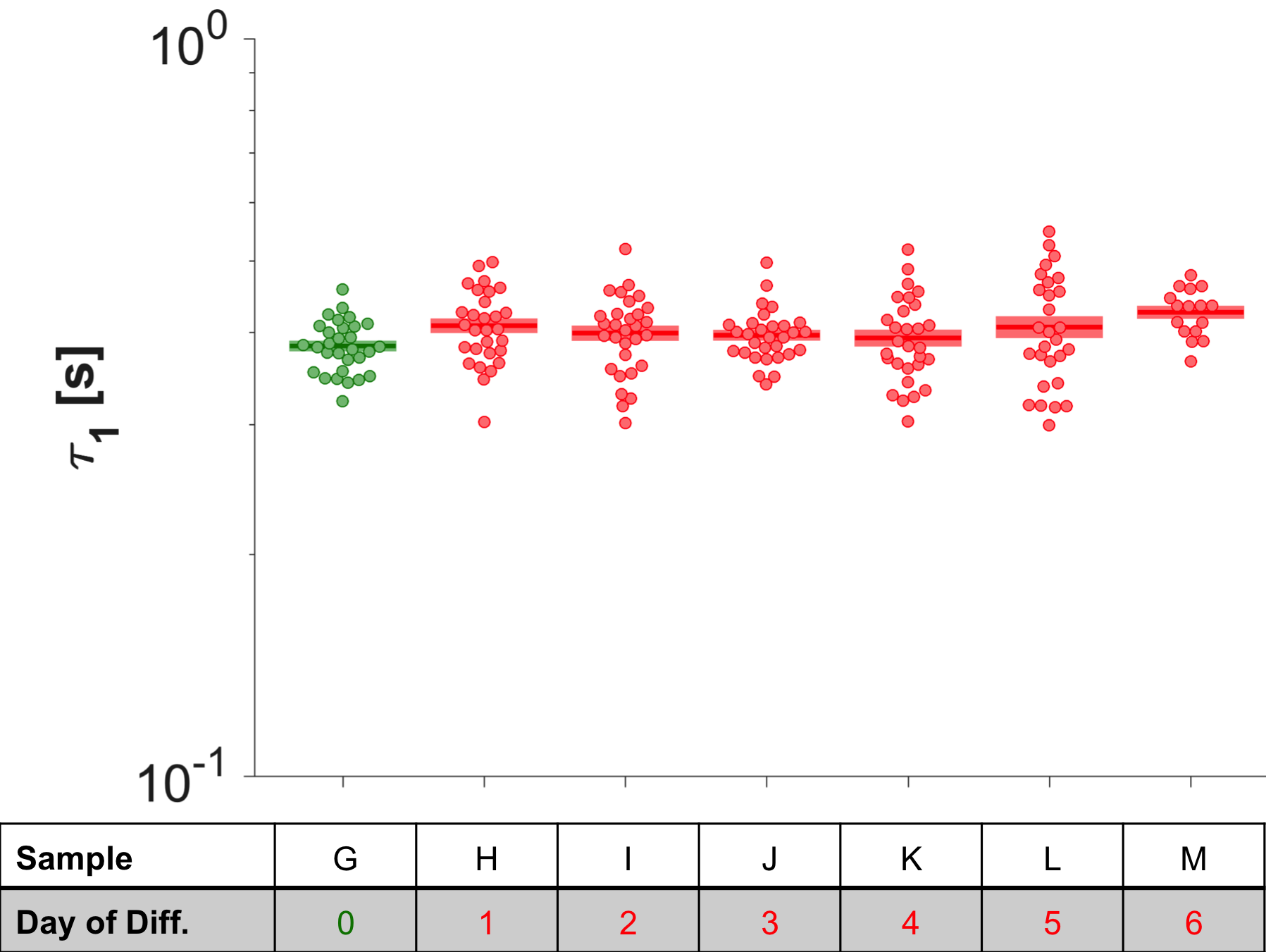

Supplement: S3 Fig — (A) Cells were stained for F-actin (fluorescent green) using phalloidin and for DNA (fluorescent blue) using Hoescht 33342. Cell morphologies were categorized as one of three types: rounded cells (left), sheet-like actin (middle), or polarized, fiber-rich actin (right). (B). As shown in the doughnut plots, the dominant morphology type changed from rounded cells (green) on days 0–1 to sheet-like actin (blue) on days 2–5 and finally to polarized, fiber-rich actin (red) on day 6. Representative images were selected from the majority morphological type for each day of differentiation. Scale bars indicate 10 μm. (TIF) [file pone.0192631.s004.tif]

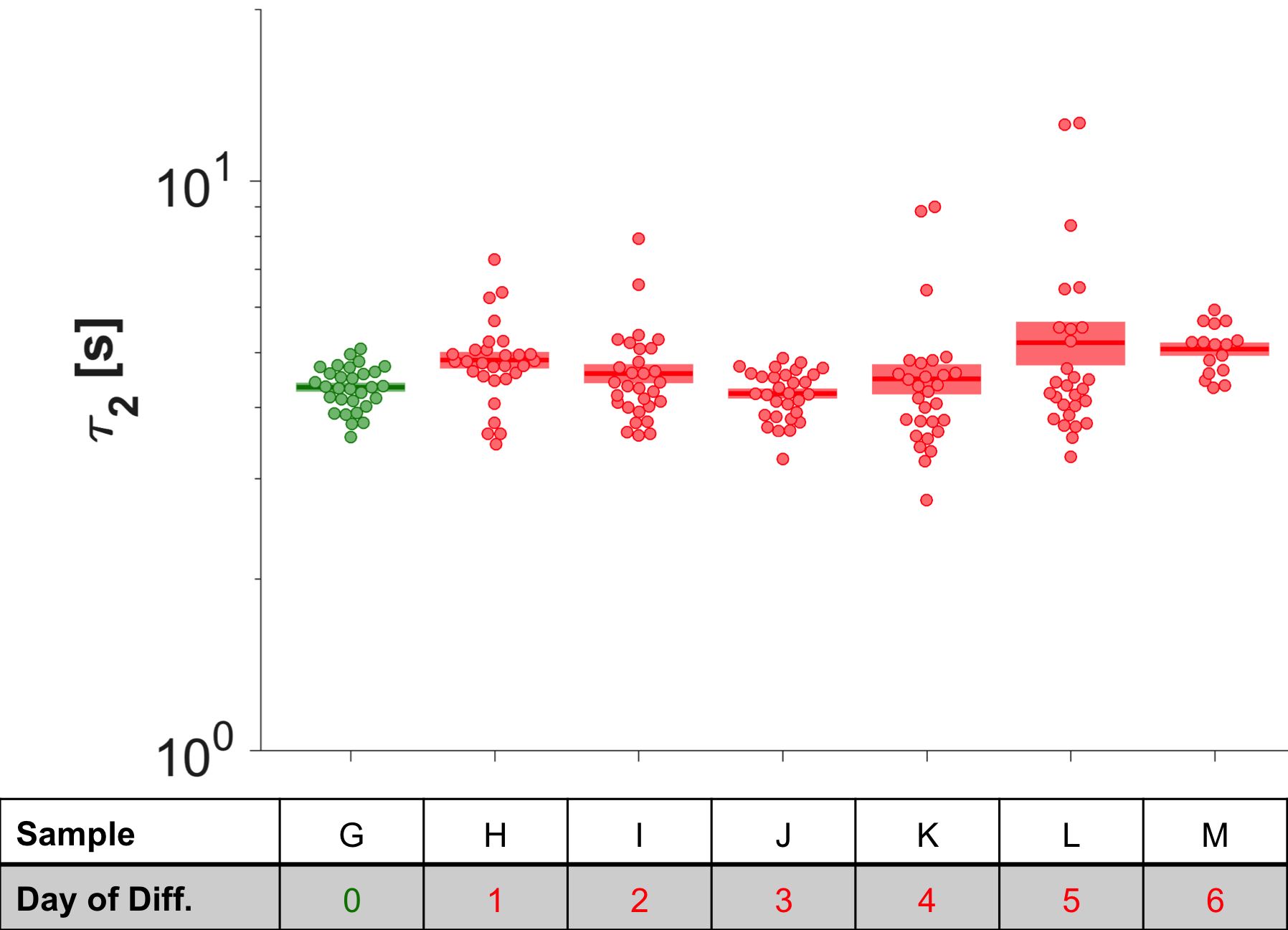

Supplement: S4 Fig — The sample letters are matched to the data in S1 Fig. For all cells, the day 0 session was 4, the day 0 passage number was 28, and differentiation was induced via leukemia inhibitory factor removal in the presence of fetal bovine serum in monolayer format. (TIF) [file pone.0192631.s005.tif]

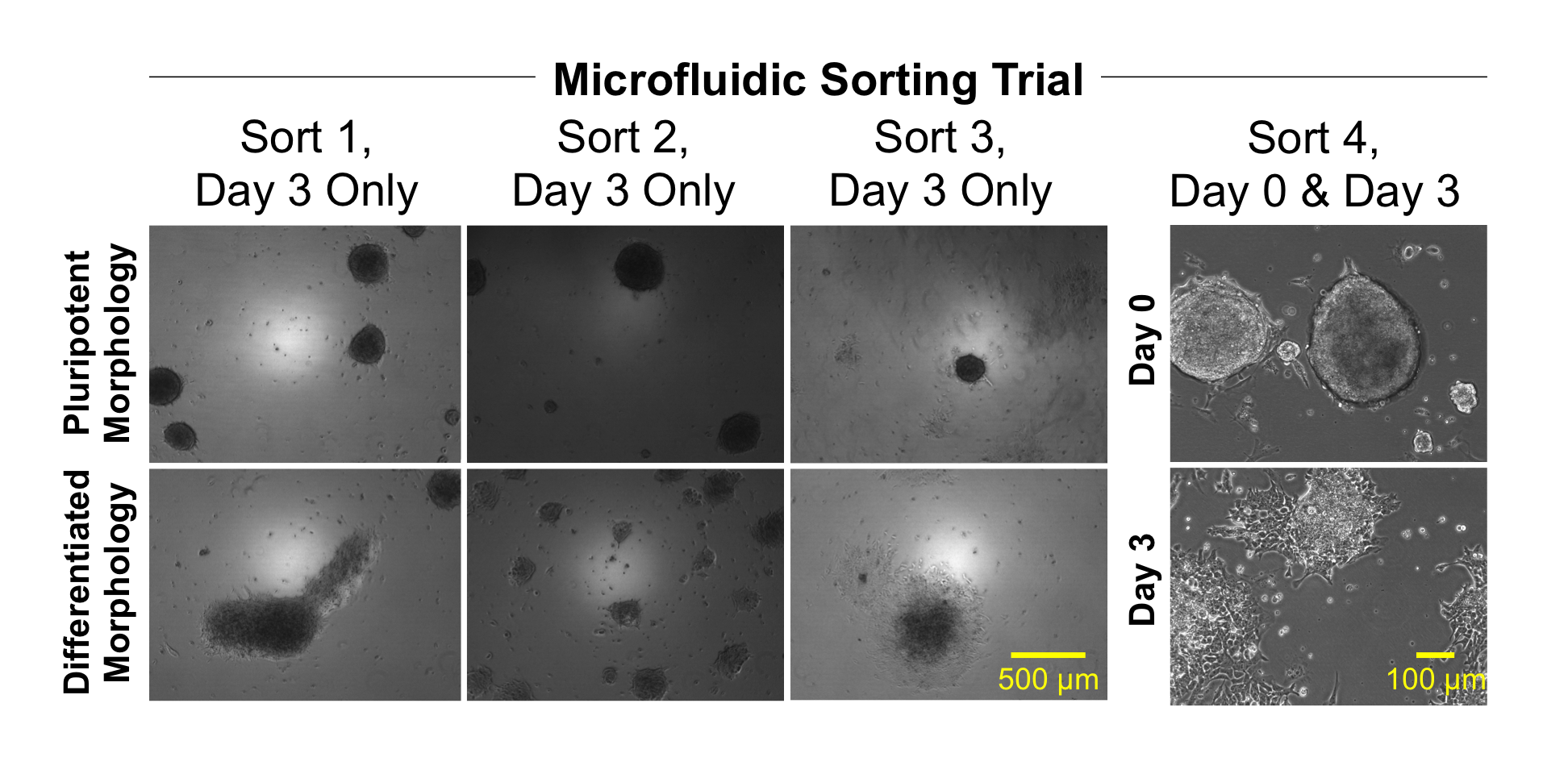

Supplement: S5 Fig — The sample letters are matched to the samples in S1 Fig. For all cells, the day 0 session was 4, the day 0 passage number was 28, and differentiation was induced via leukemia inhibitory factor removal in the presence of fetal bovine serum in monolayer format. (TIF) [file pone.0192631.s006.tif]

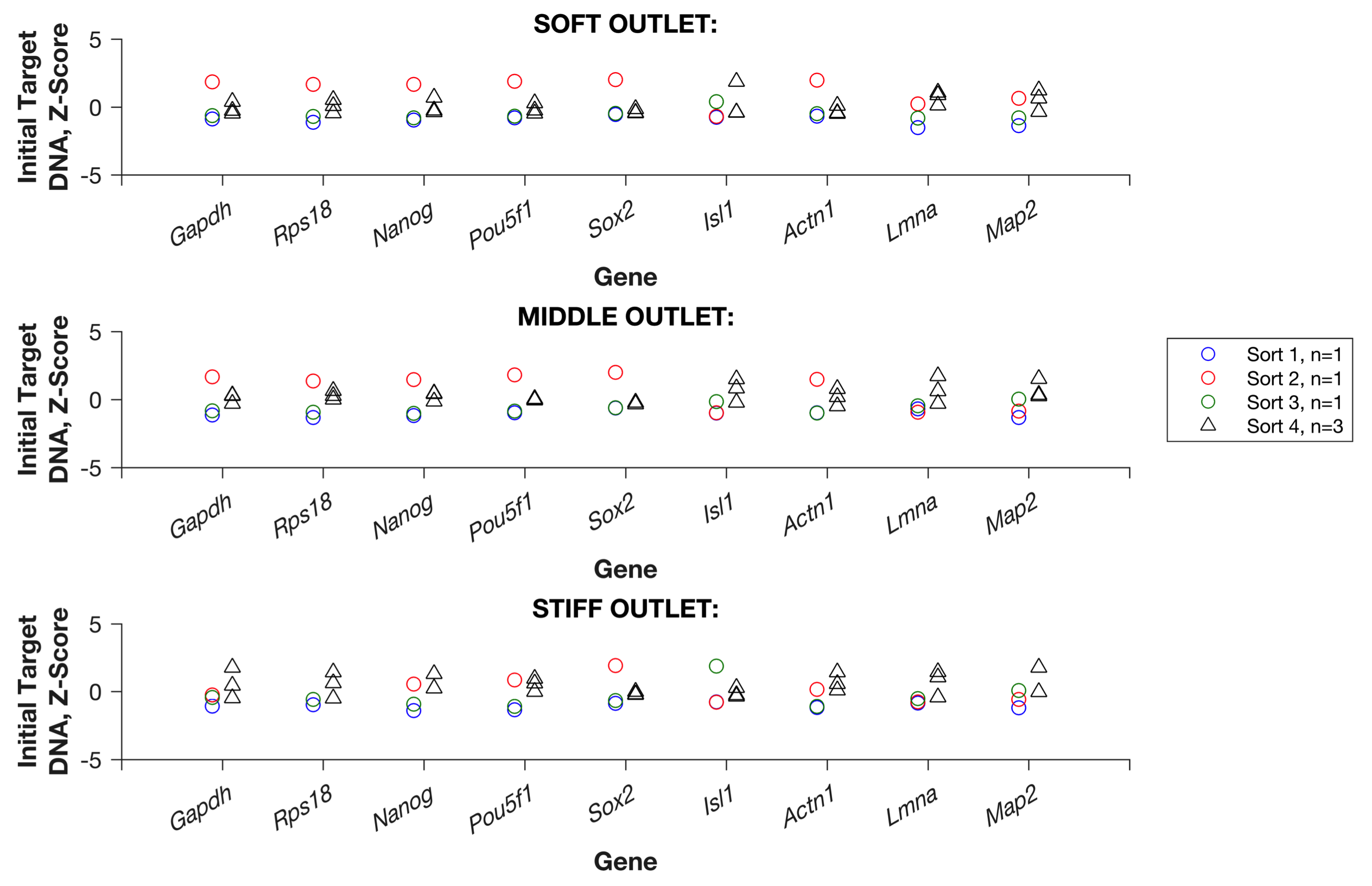

Supplement: S6 Fig — Before microfluidic sorting, the ESC cultures contained both rounded pluripotent colonies and spread differentiating colonies. For sorts 1–3, “pluripotent” and “differentiated” indicate colonies with the respective morphologies after 3 days of differentiation. For sort 4, day 0 cells with pluripotent morphology and day 3 cells with differentiated morphology were mixed prior to sorting. (TIF) [file pone.0192631.s007.tif]

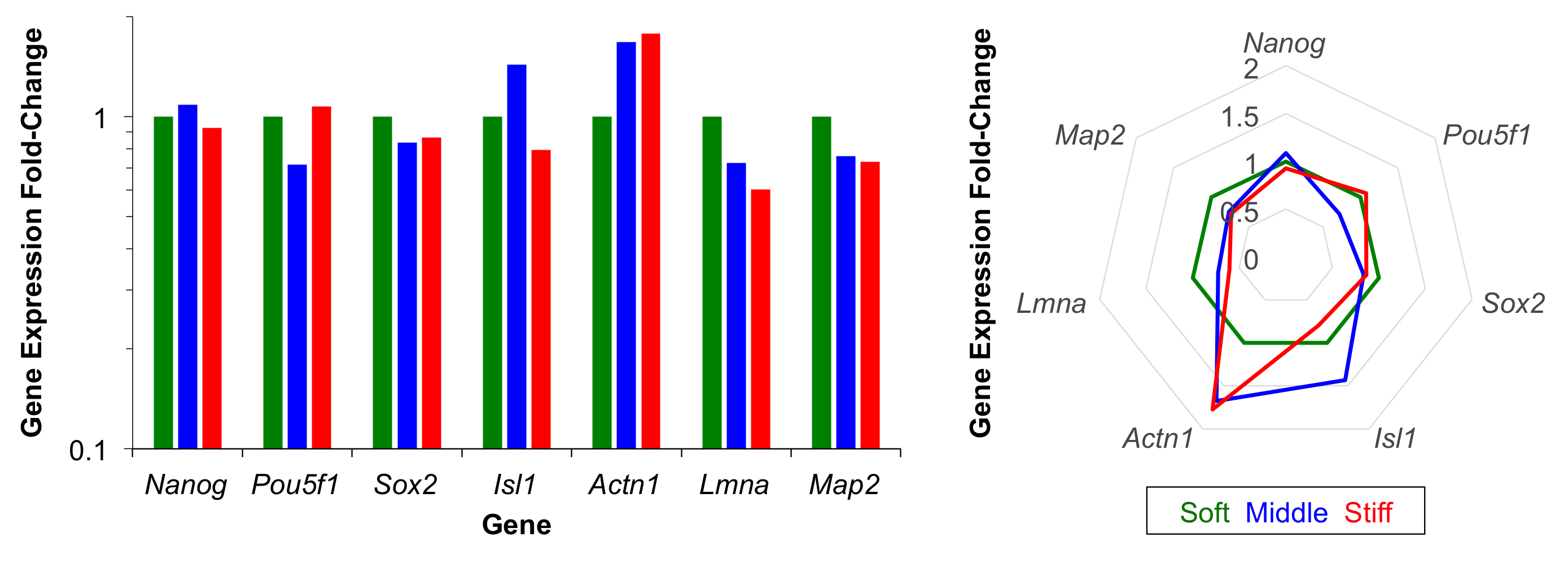

Supplement: S7 Fig — Following biophysical separation, 100-cell samples were collected for gene expression analysis. For the first 3 separation experiments (blue, red, and green circles), n = 1 100-cell replicate was collected per outlet. For the fourth separation experiment (black triangles), n = 3 100-cell replicates were collected per outlet. As the between-experiment and between-replicate initial target DNA z-scores was not substantially different, initial analysis was conducted using the pooled set of n = 6 100-cell samples (see S8 Fig). (TIF) [file pone.0192631.s008.tif]

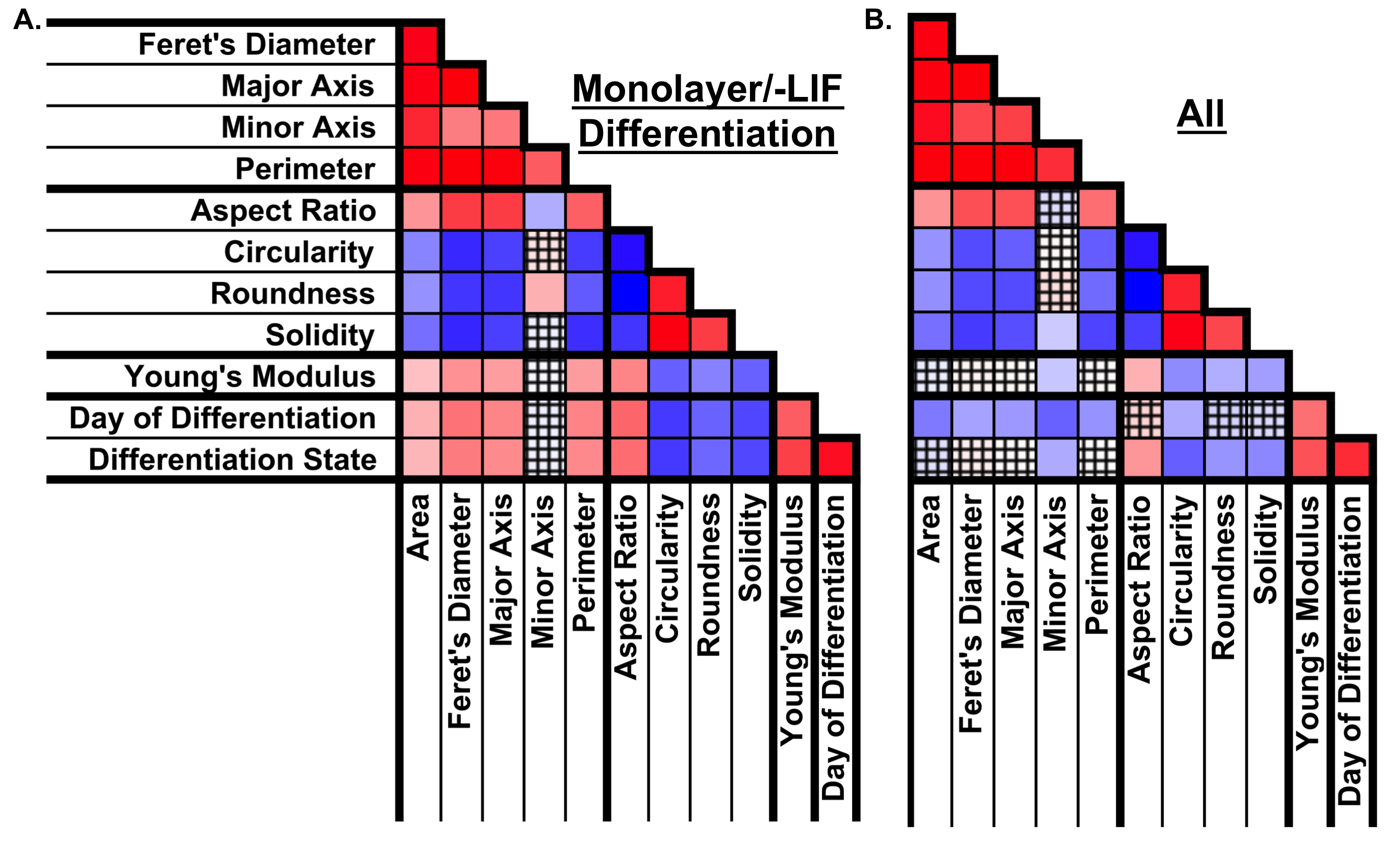

Supplement: S8 Fig — The pluripotency gene Sox2 was increased in the soft outlet, although Nanog and Pou5f1 showed unclear trends. The structural gene Actn1 increased in the middle and stiff outlets. Green, soft outlet; blue, middle outlet; red, stiff outlet; ΔΔCt values, control group = soft outlet, housekeeping gene = geometric mean of Gapdh and Rps18; mean of six 100-cell samples (see S7 Fig). (TIF) [file pone.0192631.s009.tif]

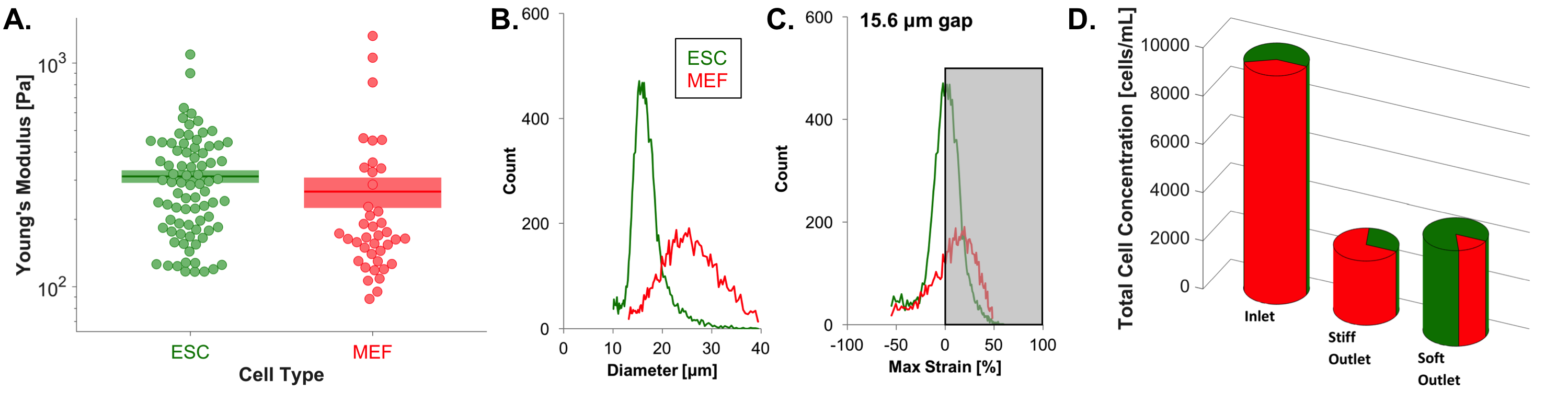

Supplement: S9 Fig — (A) There was no significant difference between the stiffness of mouse embryonic stem cells (ESCs, green) and mouse embryonic fibroblasts (MEFs, red; p = 0.329). (B) However, the cell diameter, which was measured for cells in suspension, was generally smaller for ESCs than MEFs and thus represented an independent biophysical parameter that is suitable for microfluidic sorting. (C) A 15.6 μm gap size was chosen to expose the majority of ESCs and MEFs to strain (gray shading), maximizing the differential sorting trajectory between cell types. (D) The sorting efficiencies of ESCs in the soft (small) outlet and MEFs in the stiff (large) outlet, defined similarly to eday 0 and eday 5, respectively, both exceeded 3. (TIF) [file pone.0192631.s010.tif]

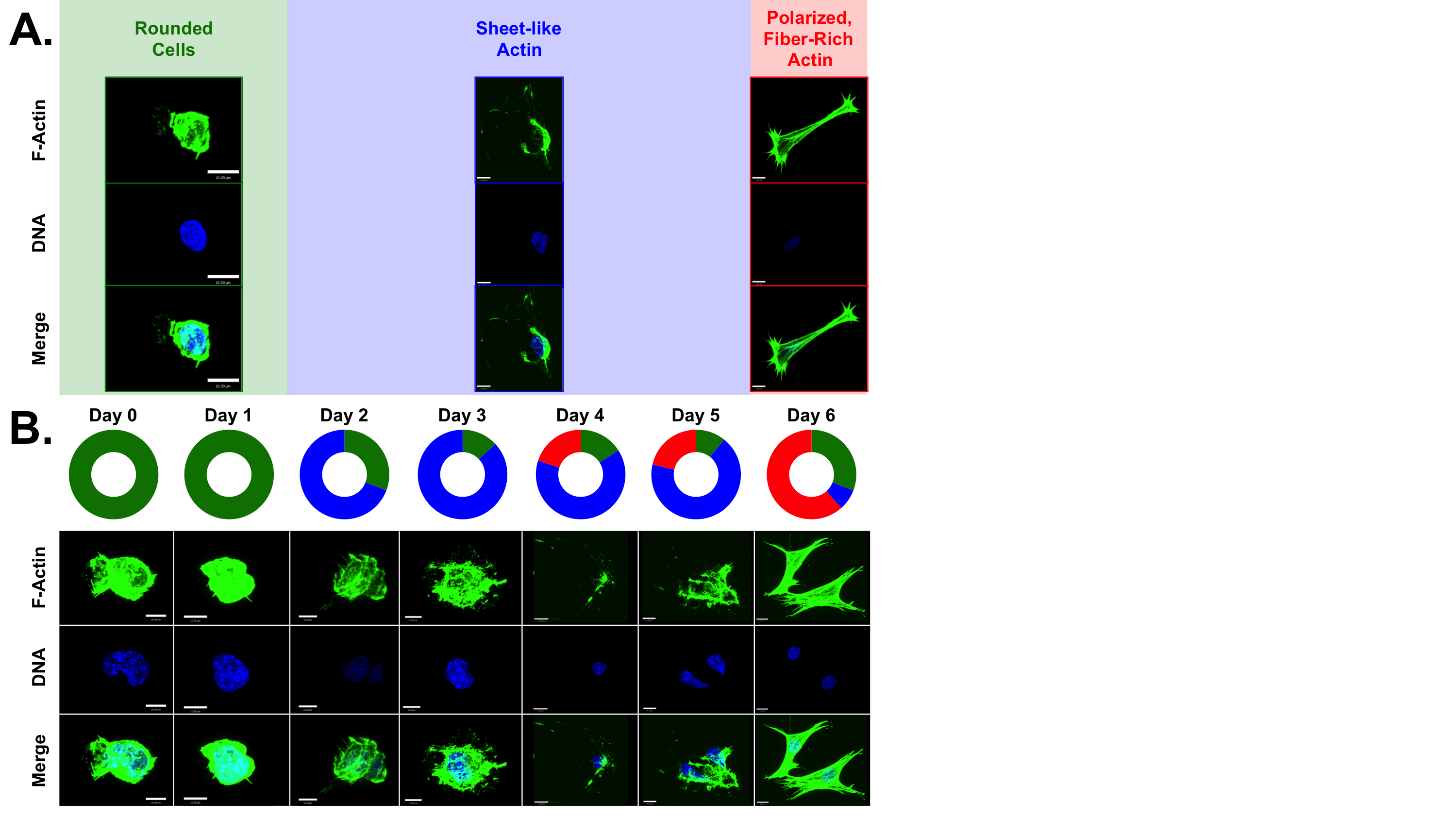

Supplement: S10 Fig — Compared to Fig 9A, similar relationships among spread cell area, spread cell roundness, mechanics, and differentiation were observed when the data set was expanded to include cells for which viscoelastic data were not available. The first data expansion included only cells differentiated in monolayer by LIF removal (A, N = 242), and the second data expansion considered all cells, including cells differentiated in embryoid body format (B, N = 359). As viscoelastic relaxation profiles were not recorded for large portions (21% and 47%, respectively) of cells in the expanded data sets, relationships with the viscoelastic relaxation time constants were not considered. (TIF) [file pone.0192631.s011.tif]
