# Supplementary material for: Biophysical subsets of embryonic stem cells display distinct phenotypic and morphological signatures
Source: PLoS One. 2018 Mar 8;13(3):e0192631. doi: 10.1371/journal.pone.0192631 (PMC5843178; doi:10.1371/journal.pone.0192631)
Supplement: S1 Table — Primers employed for pre-amplification and PCR. (DOCX) [file pone.0192631.s012.docx]

S1 Table. Primer Sequences. Primers employed for pre-amplification and PCR.

| **Gene** | **Forward Sequence (5’ to 3’)** | **Reverse Sequence (5’ to 3’)** |
| --- | --- | --- |
| *Actn1* | TTAACACGCTGCAGACCAAG | TCTCTAGCCTCCGGATCTCA |
| *Gapdh* | CATGGCCTTCCGTGTTCCTA | CCTGCTTCACCACCTTCTTGAT |
| *Isl1* | ATGATGGTGGTTTACAGGC | TCGATGCTACTTCACTGCC |
| *Lmna* | TGAGTACAACCTGCGCTCAC | TGACTAGGTTGTCCCCGAAG |
| *Map2* | AGTGGCACCTCCACACCTAC | CGGATGATGGCAACTTTCTT |
| *Nanog* | GAAATCCCTTCCCTCGCCATC | CTCAGTAGCAGACCCTTGTAAGC |
| *Pou5f1* | CCGTGTGAGGTGGAGTCTGGAG | GCGATGTGAGTGATCTGCTGTAGG |
| *Rps18* | CTCTAGTGATCCCTGAGAAGTTCC | ACTCGCTCCACCTCATCCTC |
| *Sox2* | CTCGCAGACCTACATGAACG | AGTGGGAGGAAGAGGTAACC |
